# Role of the Blood–Brain Barrier in the Pathophysiology of Major Depressive Disorder, Bipolar Disorder, and Schizophrenia: A Comparative Review

**DOI:** 10.3390/ph19091483

**Published:** 2026-09-17

**Authors:** Ira Sivan Rostevanov, Odeya Damri, Abed N. Azab, Galila Agam

**Affiliations:** 1 Department of Clinical Biochemistry and Pharmacology, Faculty of Health Sciences, Ben-Gurion University of the Negev, P.O. Box 653, Beer-Sheva 8410501, Israelgalila@bgu.ac.il (G.A.); 2 Psychiatry Research Unit at the Department of Clinical Biochemistry and Pharmacology, Faculty of Health Sciences, Ben-Gurion University of the Negev, P.O. Box 653, Beer-Sheva 8410501, Israel; 3 Department of Nursing, School for Community Health Professions, Faculty of Health Sciences, Ben-Gurion University of the Negev, P.O. Box 653, Beer-Sheva 8410501, Israel; 4 The School of Brain Sciences and Cognition, Ben-Gurion University of the Negev, P.O. Box 653, Beer-Sheva 8410501, Israel

**Keywords:** endothelial dysfunction, neuroinflammation, neurovascular unit, oxidative stress, permeability, psychiatric disorders

## Abstract

The blood–brain barrier (BBB) is a unique neurovascular interface essential for central nervous system homeostasis. Beyond its classical protective role, accumulating evidence points to the BBB as a dynamic structure actively involved in brain function in health and disease. The present review synthesizes clinical, molecular, and neuroimaging evidence implicating BBB dysfunction in major depressive disorder (MDD), bipolar disorder (BD), and schizophrenia. The paper was prepared in accordance with the recommendations of the Scale for the Assessment of Narrative Review Articles and was based on a PubMed search of publications published between 2000 and 2026. We summarize BBB architecture and regulation within the neurovascular unit and examine how neuroinflammation, oxidative stress, mitochondrial dysfunction, vascular-metabolic disturbances, and glucocorticoid signaling compromise tight junction, endothelial, pericyte, and astrocytic function, thereby facilitating peripheral immune signaling and sustaining central inflammation. Disorder-specific findings highlight shared and distinct mechanisms: In MDD, EZH2-mediated downregulation of claudin-5 and VEGF-driven barrier disruption are associated with increased permeability in the prefrontal cortex and hippocampus; in BD, elevated cerebrospinal fluid/serum albumin ratios and levels of S100B and matrix metalloproteinase-9-mediated tight junction degradation are associated with illness duration and neuroprogression; and in schizophrenia, reduced expression of claudin-5 and claudin-11, diminished pericyte coverage, and dysregulation of transforming growth factor-α/platelet-derived growth factor signaling are associated with neuroinflammation and treatment resistance. Therapeutic implications are discussed, including anti-inflammatory and antioxidant strategies, endothelial stabilization, and BBB-targeted delivery, as well as methodological limitations and future directions for biomarker development and neurovascular modeling.

## 1. Introduction

### 1.1. The BBB and Its Role in Maintaining Central Nervous System Homeostasis

The blood–brain barrier (BBB) is a highly specialized, selective semipermeable membrane that separates the circulating blood from the brain and extracellular fluid in the central nervous system (CNS) [1]. It is primarily composed of endothelial cells connected by tight junctions (TJs)—multiprotein complexes that seal the intercellular gaps—supported by astrocytes, pericytes, and the extracellular matrix, together forming the neurovascular unit [2]. By tightly regulating molecular exchange between blood and the brain, the BBB maintains CNS homeostasis, preserves ionic balance, shields neural tissue from peripheral immune responses, and supports proper neurotransmission [3,4]. Conversely, loss of BBB integrity permits uncontrolled entry of peripheral inflammatory mediators, immune cells, and neurotoxic compounds, and has therefore been implicated in neuroinflammation and in the progression of neurological illnesses, including psychiatric disorders [4,5,6].

### 1.2. Cellular Composition of the BBB

**Figure 1:**
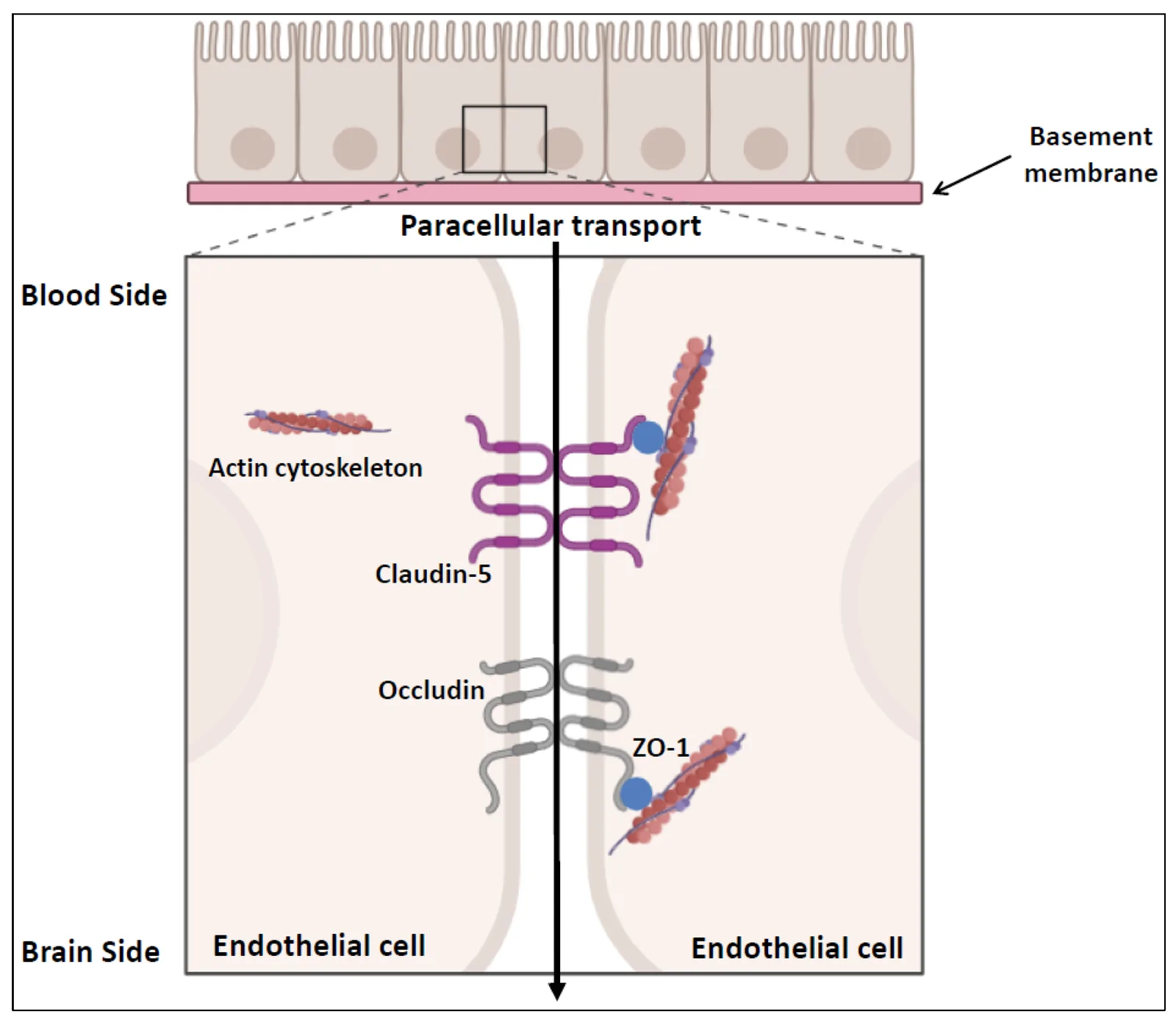
An intricate protein network creates the BBB. Created in BioRender. Rostevanov, IS. (2026) https://BioRender.com/3hr7mnd (accessed on 4 August 2026).

Unlike endothelial cells in peripheral vessels, the brain microvascular endothelial cells lining cerebral capillaries exhibit minimal pinocytotic activity, lack fenestrations, express specific transporters regulating molecular exchange, and are sealed by intercellular TJ complexes that restrict paracellular diffusion [7,8]. These TJs consist of transmembrane proteins, such as claudin-5, occludin, and junctional adhesion molecules (JAMs), which interact with cytoplasmic scaffold proteins including zonula occludens (ZO)-1, ZO-2, and ZO-3 to anchor the junctional complex to the actin cytoskeleton and maintain the structural cohesion and low permeability characteristic of the BBB (Figure 1) [8,9].

Pericytes, embedded within the basement membrane and wrapped around capillaries and small blood vessels, contribute to vessel stability, angiogenesis, and regulation of BBB permeability through platelet-derived growth factor receptor (PDGFR)-β, transforming growth factor (TGF)-β, and angiopoietin/Tie (Ang/Tie) signaling [5]. Astrocytic endfeet cover over 90% of the capillary surface and serve several roles: they (i) provide structural support to the cerebral vasculature; (ii) maintain endothelial TJs, which restrict the passage of large molecules from the bloodstream into the brain; (iii) mediate neurovascular coupling by relaying signals from neurons to the vasculature to regulate blood flow; (iv) serve as a major site for the exchange of ions, metabolites, and energy substrates between the blood and the brain; and (v) release vasoactive molecules that signal to pericytes, thereby regulating capillary diameter and cerebral blood flow. They further release paracrine factors such as glial-derived neurotrophic factors (GDNF), angiopoietins, and vascular endothelial growth factors (VEGF) that sustain endothelial function and TJ integrity [10]. Together with neurons, microglia, and the extracellular matrix, these components constitute the neurovascular unit (NVU), the disruption of any single element of which can compromise BBB integrity and lead to neuroinflammatory and/or neurodegenerative consequences [11,12].

### 1.3. Physiological Roles of the BBB

Several specialized systems support the homeostatic function of the BBB. Efflux transporters, notably P-glycoprotein (P-gp) and multidrug resistance-associated proteins (MRPs), protect the brain by actively exporting xenobiotics and metabolic by-products [13,14], whereas enzymatic barriers such as endothelial monoamine oxidases and cytochrome P450 enzymes degrade circulating neurotoxic molecules before they reach neural tissue [15,16]. Moreover, the BBB plays a critical role in neuroimmune regulation, preventing uncontrolled infiltration of immune cells into the CNS while allowing selective lymphocyte trafficking under physiological conditions [17]. This tight control sustains the ionic and neurotransmitter gradients required for synaptic plasticity and cognitive function. For example, concentrations of the excitatory neurotransmitter glutamate in brain extracellular fluid (ECF) are 10 to 100 times lower than in plasma, a gradient maintained by highly restricted passage across the BBB together with active transport systems that remove excess glutamate from the brain ECF to the peripheral circulation [18,19].

### 1.4. Mechanisms of BBB Regulation

BBB integrity is dynamically maintained by a complex interplay of signaling pathways that regulate endothelial stability, junctional protein expression, and interactions within the NVU [8]. Wnt/β-catenin signaling promotes BBB differentiation and upregulates TJ proteins [20], and astrocyte-derived Sonic Hedgehog (Shh) signaling enhances barrier integrity by inducing TJ components and anti-inflammatory molecules in endothelial cells [21,22,23]. The VEGF and Ang/Tie2 pathways exert dual effects: while physiological VEGF signaling supports vascular maintenance, excessive VEGF or Ang2 activation can increase permeability and endothelial stress [24,25,26,27]. Glucocorticoid signaling is similarly bidirectional: it transcriptionally induces claudin-5 and occludin [28,29]; however, excessive signaling can impair barrier function and increases permeability [30,31]. Endothelial cells are also sensitive to oxidative stress and inflammatory cytokines such as tumor necrosis factor (TNF)-α, interleukin (IL)-6, and interferon (IFN)-γ, which activate nuclear factor (NF)-κB and mitogen-activated protein kinase (MAPK) pathways, leading to junctional disruption and enhanced paracellular leakage [32]. Conversely, pericyte–endothelial crosstalk via PDGF-BB/PDGFR-β and TGF-β stabilizes the vascular basement membrane and prevents permeability increases [27,33,34].

### 1.5. Rationale and Aim of the Review

Major depressive disorder (MDD), bipolar disorder (BD), and schizophrenia are among the most disabling mental disorders. In 2023, an estimated 236 million, 35.7 million, and 26.9 million people worldwide were living with these conditions, respectively, and mental disorders as a group were the leading cause of years lived with disability globally [35]. Accordingly, these disorders affect a substantial proportion of the general population; for example, a 2002 study reported lifetime prevalences of approximately 10%, 1.5%, and 1% for MDD, BD, and schizophrenia, respectively [36]. A recent study reported similar or even higher estimates: 15.5% for MDD, 1.5% for BD, and 1.2% for schizophrenia [37].

Although BBB regulatory mechanisms have been characterized primarily in neurological conditions such as stroke and neurodegenerative diseases [5], converging neuroimaging, cerebrospinal fluid biomarker, and postmortem evidence indicates that comparable, albeit subtler, barrier impairments occur in major psychiatric disorders [38,39,40,41,42,43]. However, this evidence has largely accumulated within separate diagnostic categories and has rarely been examined comparatively, leaving unclear which neurovascular alterations are shared and which are disorder-specific. Therefore, this review integrates current clinical, imaging, and molecular evidence on BBB dysfunction across these three disorders, identifies key knowledge gaps, and explores BBB-directed therapeutic strategies as a novel approach to treating psychiatric disorders.

## 2. Methods

This article is a narrative review which was prepared in accordance with the recommendations of the Scale for the Assessment of Narrative Review Articles (SANRA) [44]. A narrative approach was considered appropriate given the broad and heterogeneous nature of the literature on BBB dysfunction in psychiatric disorders, which encompasses molecular, preclinical, neuroimaging, and clinical studies. The literature was thematically organized according to the conceptual structure of the review, allowing shared and disorder-specific mechanisms to be synthesized and compared across MDD, BD, and schizophrenia. Key studies are summarized in Tables 1–3; these include clinical, post-mortem, preclinical and in vitro investigations, whereas narrative reviews cited in the text are not tabulated.

### 2.1. Search Strategy

The literature search was conducted primarily using the PubMed database and covered publications from 2000 to 2026, with the exception of a small number of (additional) seminal earlier papers cited for conceptual context. The search was last updated on 1 September 2026. The following keywords were used both individually and in combination: ‘blood-brain barrier’, ‘BBB permeability’, ‘neurovascular unit’, ‘tight junctions’, ‘claudin-5’, ‘pericytes’, ‘endothelial dysfunction’, ‘neuroinflammation’, ‘oxidative stress’, ‘major depressive disorder’, ‘bipolar disorder’, and ‘schizophrenia’. In addition to the primary database search, the reference lists of retrieved articles and of relevant reviews were screened manually to identify further eligible publications. Eligible sources included seminal papers, experimental and preclinical investigations, clinical studies, neuroimaging studies, and meta-analyses published in peer-reviewed journals in English. Conference abstracts, non-peer-reviewed preprints, and publications in languages other than English were excluded, as were studies confined to non-psychiatric neurological conditions without a mechanism relevant to the scope of this review. Selection was based on relevance to the central theme of BBB dysfunction in psychiatric disorders, with priority given to original articles and to reports providing mechanistic, imaging, or biomarker-level evidence. Records were screened independently by the authors, with any disagreement resolved by discussion. A total of 261 publications were included in the final synthesis, of which 210 (80%) were published in 2020 or later. The principal studies, together with their design, populations or models, and the barrier markers examined, are presented in Tables 1–3.

### 2.2. Study Quality Assessment

No formal risk-of-bias assessment was performed because quantitative pooling was not undertaken and the included studies encompassed diverse designs for which no single appraisal tool was appropriate. Sources were instead appraised qualitatively and weighted according to a design hierarchy: meta-analyses and systematic reviews were given the greatest weight, followed by controlled clinical and neuroimaging studies, uncontrolled clinical and post-mortem series, in vivo preclinical models, and in vitro systems. Within the clinical literature, additional weight was given to studies employing direct and objective indices of barrier integrity—dynamic contrast-enhanced magnetic resonance imaging (DCE-MRI), the cerebrospinal fluid/serum albumin quotient, and post-mortem quantification of tight junction proteins—over peripheral surrogate markers alone. Sample size, presence of a control group, control for relevant confounders (including psychotropic medication, age, sex, smoking, and comorbid metabolic disease), and independent replication were considered when assessing the strength of individual claims. Findings supported by a single study, or derived exclusively from preclinical models, are identified as preliminary in the text and are not presented as established mechanisms in humans. Where evidence is cross-sectional, associations are described as such rather than as causal relationships.

## 3. BBB Dysfunction and Psychiatric Disorders

Current models view BBB disruption in psychiatric disorders not as a secondary consequence of chronic illness or medication exposure, but as an active contributor to disease pathophysiology. As described below, four partially overlapping processes affect the NVU: neuroimmune activation, oxidative and mitochondrial stress, vascular-metabolic dysregulation, and stress-mediated endothelial damage. Once BBB integrity is compromised, the influx of peripheral mediators can sustain the processes that caused the disruption, creating a self-reinforcing cycle that may contribute to symptom persistence, treatment resistance, and disease progression. The following subsections discuss each of these mechanisms.

**Figure 2:**
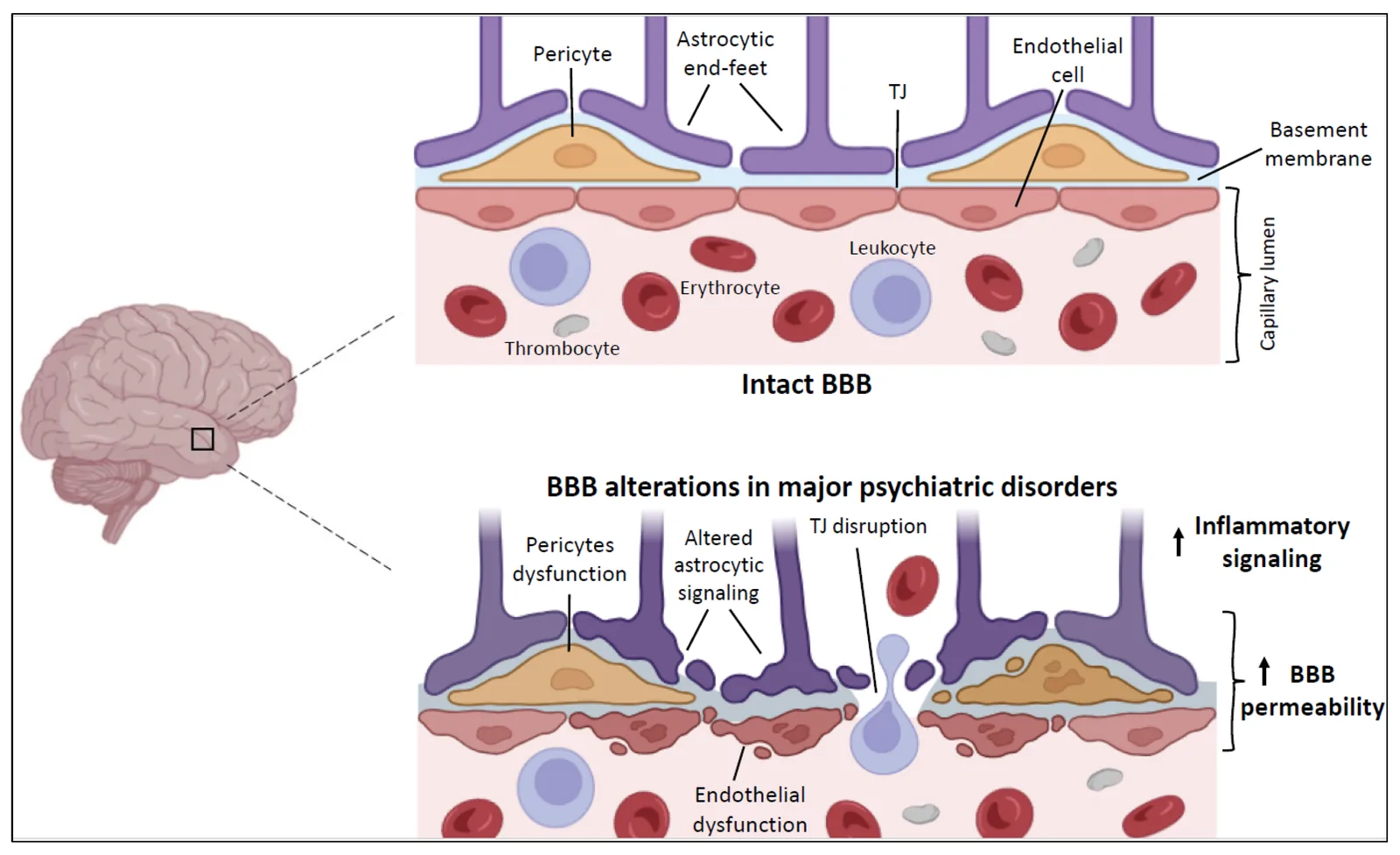
Structural and functional alterations of the BBB in major psychiatric disorders. The upper panel depicts an intact BBB under physiological conditions. The lower panel illustrates BBB alterations reported in major psychiatric disorders including increased BBB permeability, inflammatory-related vascular changes, tight junction (TJ) disruption, pericyte dysfunction, endothelial cell dysfunction and altered astrocytic end-feet signaling. Arrow—denotes an increase. Created in BioRender. Rostevanov, IS. (2026) https://BioRender.com/oteqeqt (accessed on 4 August 2026).

Figure 2 summarizes the structural and functional alterations reported across MMD, BD, and schizophrenia. For example, increased permeability has been observed in specific brain regions among patients with schizophrenia [38,40]. These findings suggest that BBB dysfunction may permit influx of peripheral immune cells and inflammatory cytokines, thereby inducing neuroinflammatory cascades that interfere with neurotransmitter balance and neural circuit stability. Furthermore, endothelial dysfunction, oxidative stress, and reactive glial activation, which perpetuate BBB breakdown [6,45,46,47], may not only exacerbate ongoing psychiatric symptoms but also contribute to treatment resistance and disease chronicity.

As mentioned above, claudin-5, occludin, and ZO-1 are proteins that form TJs. Reduced expression of claudin-5 has been reported in the hippocampus of patients with depression and schizophrenia, as well as in stress-exposed rodent models showing downregulation of claudin-5, occludin, and ZO-1 in frontal and limbic regions [48,49,50]. Importantly, BBB dysfunction appears to interact bidirectionally with psychiatric pathology as follows: stress, altered glucocorticoid signaling, and systemic inflammation might impair BBB integrity, while barrier disruption itself may reinforce maladaptive neural processes. 

### 3.1. Neuroinflammation and Immune Activation

Neuroinflammation is both a cause and a consequence of BBB disruption [51]. Peripheral inflammatory mediators (e.g., IL-1β, TNF-α, IL-6) and activated innate immune receptors expressed by cerebral endothelial cells (such as toll-like receptors) trigger endothelial signaling cascades, NF-κB and MAPK pathways, which downregulate TJ proteins and increase paracellular permeability [51,52,53,54,55]. Beyond these canonical pathways, the NLRP3 inflammasome and the kynurenine pathway may also link immune activation to neuronal dysfunction. The latter can shift tryptophan metabolism away from serotonin synthesis toward quinolinic acid production, thereby promoting excitotoxicity [56]. Experimental studies in animal models and endothelial cell cultures, alongside clinical observations in humans, show that systemic inflammation rapidly alters endothelial transcription programs and transporter function, promoting leukocyte adhesion and diapedesis (transmigration) across the barrier [32]. This influx of peripheral immune cells and cytokines amplifies microglial activation and sustains a local inflammatory milieu that feeds back to further degrade TJ complexes and basement membrane integrity [53,57]. Thus, neuroinflammation generates a vicious cycle in which BBB opening facilitates immune access and activation, further impairing the barrier’s function [58,59,60,61]. 

Evidence supporting this cascade of events comes from multiple levels. In clinical psychiatric cohorts, for example, patients with mood or psychotic disorders, elevated peripheral and CSF inflammatory markers have been observed alongside imaging or biomarker indications of BBB leakiness [41,62,63,64,65,66]. Meta-analyses directly comparing the three disorders describe overlapping but non-identical cytokine profiles, with IL-6 and TNF-α elevated during acute episodes of MDD, BD and schizophrenia [67], and low-grade inflammation is present in a substantial proportion of patients with MDD [68]. Importantly, circulating CRP, IL-6 and TNF-α have been associated not only with diagnosis but with greater symptom severity and poorer response to conventional pharmacotherapy [56]. Within the CSF, comparable elevations of IL-1β, IL-6, IL-8 and kynurenine metabolites have been reported across these disorders, in some cohorts accompanied by concurrent evidence of BBB dysfunction, although the underlying studies are heterogeneous and of variable quality [69,70]. Consistently, postmortem studies of the midbrain have found inflammation-related angiogenesis and endothelial changes associated with macrophage migration across the vessel wall in schizophrenia and bipolar disorder, particularly in individuals with high levels of inflammation [71].

Evidence that inflammation may contribute to psychiatric disorders, rather than simply result from them, comes from several lines of research. In humans, bidirectional Mendelian randomization studies have found that genetically higher IL-6 levels are associated with a greater risk of MDD and schizophrenia [72]. In male mice, chronic social defeat stress reduced claudin-5 expression in the nucleus accumbens of stress-susceptible, but not stress-resilient, mice [73]. Importantly, viral knockdown of claudin-5 was sufficient to induce depression-like behavior following subthreshold stress and increase the infiltration of peripheral IL-6 into the brain parenchyma; both effects were reversed by chronic antidepressant treatment [73]. A similar pattern has been observed in female mice, in which stress-induced loss of claudin-5 in the prefrontal cortex was associated with anxiety- and depression-like behavior [74]. The temporal sequence has been examined directly in a chronic infection model, in which pro-inflammatory cytokine and chemokine levels increased before the onset of neuroinflammation abnormalities [75]. This was followed by BBB disruption and the development of anxiety, depressive-like behavior, and hyperactivity. These behavioral changes persisted despite a progressive decline in parasite burden, suggesting that neuroinflammation, rather than the infectious stimulus itself, was the most consistent correlate of behavioral abnormalities [75]. Complementing these findings, experimental models (both in vivo rodent models and in vitro BBB/NVU systems) demonstrate that blocking key inflammatory mediators can attenuate barrier disruption [41,66,76,77,78].

### 3.2. Oxidative Stress and Mitochondrial Dysfunction

Oxidative stress is a central mediator of BBB injury [45]. Reactive oxygen species (ROS) generated by dysfunctional mitochondria, activated immune cells, and/or dysfunctional metabolic pathways oxidize lipids, proteins, and nucleic acids of endothelial and perivascular cells [45,79,80]. At the BBB, ROS promote phosphorylation and internalization of TJ proteins and destabilize adherence junctions, increasing paracellular leakage [45,79,80]. Mitochondrial dysfunction in brain microvascular endothelial cells reduces the energy supply required for ion pumps and junctional maintenance, rendering the barrier more susceptible to inflammatory and hemodynamic stressors [81]. Mechanistically, oxidative stress engages redox-sensitive kinases and proteases, notably matrix metalloproteinases (MMPs), that cleave extracellular matrix components and TJ-associated proteins, further weakening the NVU scaffold [80]. Importantly, oxidative and inflammatory pathways are tightly coupled: cytokine signaling increases ROS production, while oxidative damage augments inflammatory gene expression, creating a feed-forward loop that sustains BBB dysfunction [82].

Evidence that these processes are relevant to psychiatric disorders comes from both clinical and experimental sources. Meta-analytic data from patients with BD indicate elevated levels of lipid peroxidation markers and nitrites, together with reduced glutathione levels, with lipid peroxidation elevated in both manic and depressive states [83]. In schizophrenia, impaired antioxidant defenses have been reported, with some alterations serving as potential state markers of acute exacerbation, whereas others may represent trait markers [84]. Similarly, increased oxidative damage to DNA and lipids has been reported in MDD [85]. Notably, these peripheral abnormalities are largely shared across the three disorders, whereas post-mortem studies suggest a degree of disorder-specificity at the mitochondrial level. For example, complex I activity and levels of its NDUFS7 subunit have been reported to be reduced in the prefrontal cortex of patients with BD, but not in those with MDD or schizophrenia, whereas 3-nitrotyrosine, a marker of protein nitration, was elevated in both BD and schizophrenia [86]. However, it is important to emphasize that findings across brain regions and cohorts have not been entirely consistent. Experimental studies directly link these abnormalities to impaired barrier function: in brain microvascular endothelial cells, Drp1–Fis1-mediated mitochondrial fission was accompanied by loss of TJ proteins and increased permeability, while disruption of this interaction attenuated barrier failure [87]. On the other hand, in a hypoxia model, inhibition of mitochondrial ROS production prevented the reduction in ZO-1 and occludin and the accompanying increase in permeability, an effect observed in senescent human brain endothelial cells and in aged (but not young) mice [88]. 

Together, these findings position oxidative and mitochondrial disturbances not merely as correlates of psychiatric illness, but as plausible mechanisms through which they compromise barrier integrity in these disorders.

### 3.3. Vascular and Metabolic Factors 

Vascular dysfunction and metabolic derangements substantially influence BBB integrity [47,89]. Endothelial dysfunction, manifested by impaired nitric oxide signaling, endothelial activation, and dysregulated angiogenic factors, alter the structural and functional properties of cerebral micro-vessels [27,90]. While physiological VEGF signaling supports angiogenesis and vessel maintenance, excessive VEGF or Ang-2 function destabilizes the endothelium, increases permeability, and promotes leukocyte trafficking. Moreover, metabolic factors such as hyperglycemia and dyslipidemia modulate endothelial resilience by enhancing oxidative stress and increasing MMP activity and basement membrane degradation [91,92,93].

Using DCE-MRI—the reference standard for quantifying barrier leakage in vivo—increased BBB permeability was detected in a subset of patients with BD and was associated with higher body mass index, insulin resistance, and cardiovascular disease; greater BBB permeability was associated with a more severe illness course [94]. Consistent with a vascular component, cerebral blood flow studies in BD indicate hypoperfusion, particularly in frontal regions, during mood episodes [95]. These findings provide a direct link between systemic metabolic comorbidities, which are common in BD and MDD, and barrier vulnerability, while suggesting that only a subgroup of patients may be affected [93].

### 3.4. Stress, Glucocorticoids, and Endothelial Damage

Psychological and physiological stressors influence BBB function through neuroendocrine and direct endothelial signaling [28,96,97]. Glucocorticoids (GCs) exert context-dependent effects on the BBB: acute GC signaling can promote TJ transcription and transiently strengthen barrier function, an effect mediated by direct glucocorticoid receptor binding to a response element in the occludin promoter [98,99], whereas chronic or dysregulated exposure alters the transcription, expression, and localization of claudin-5 and occludin, compromising junctional integrity and increasing permeability [100,101]. 

These processes have been demonstrated directly in models of stress-related pathology. Chronic social defeat stress reduced claudin-5 expression in the nucleus accumbens of stress-susceptible male mice and increased infiltration of peripheral IL-6, with both changes reversed by chronic antidepressant treatment [73]. Similar findings have been described in the prefrontal cortex of female mice, where they were accompanied by anxiety- and depression-like behaviors, with corresponding reductions in CLDN5 expression observed in post-mortem tissue from patients with depression [74]. In humans, disruption of tight-junction components has been reported as a shared feature across major psychiatric disorders [50]. Notably, these alterations vary according to the stress paradigm, brain region, and sex, and distinguish stress-susceptible from resilient animals rather than simply reflecting stress exposure itself [74]. Thus, stress and GC signaling provide a mechanistic route by which psychosocial factors can translate into neurovascular and immune changes relevant to psychiatric pathophysiology.

## 4. Pathophysiological Mechanisms of Specific Psychiatric Disorders

Psychiatric disorders represent one of the most significant public health challenges of our time, inflicting profound suffering on millions of individuals globally and placing an extraordinary burden on healthcare systems and economic resources [35]. The pathophysiology of major psychiatric disorders is multifactorial, involving complex interactions among genetic, molecular, immune, and environmental factors that disrupt brain function and behavior [102,103,104,105]. Despite their clinical differences, all three disorders have been associated with BBB abnormalities. The subsections below examine the evidence for each disease, distinguishing findings obtained in patients from those derived from preclinical models.

### 4.1. Major Depressive Disorder (MDD)

MDD, a particularly prevalent mental disorder [35], is characterized by a persistent state of low mood that often arises without an identifiable direct cause [106,107,108]. MDD presents with symptoms extending beyond mood disturbances, including psychological distress, diminished self-worth, changes in appetite and sleep, anhedonia, social withdrawal, reduced libido, and thoughts of self-harm and suicide [106,107,108]. Although depression has traditionally been conceptualized in terms of monoaminergic deficits, converging evidence implicates the neurovascular interface, with chronic stress and dysregulated GC signaling acting not merely as systemic consequences but as potential drivers of neurovascular dysfunction that contribute to depressive phenotypes.

Evidence from patients. Recent clinical evidence using DCE-MRI has demonstrated significantly increased BBB permeability in the prefrontal cortex and hippocampus of MDD patients, suggesting that barrier leakage is a core feature of the disorder pathogenesis [48]. This is further supported by post-mortem studies showing a marked reduction in the expression of crucial TJ proteins, specifically claudin-5 and occludin, which directly correlates with increased neuroinflammation [73,74]. Reduced CLDN5 expression has likewise been documented in the nucleus accumbens of individuals who died during a depressive episode [73]. In the periphery, elevated plasma levels of claudin-5 have recently been identified as a potential biomarker of peripheral inflammatory activity and BBB disruption in patients with MDD [109].

Evidence from preclinical models. The most direct evidence that loss of claudin-5 contributes causally to depressive phenotypes comes from chronic social defeat stress models in which claudin-5 is selectively reduced in the nucleus accumbens of stress-susceptible, but not resilient, mice [73]. Knockdown of claudin-5 in this region was sufficient to induce a depression-like phenotype following subthreshold stress, permit infiltration of peripheral IL-6 into the parenchyma, and produce changes that were reversed by chronic antidepressant treatment [73]. The causal role of BBB dysfunction in MDD has been further elucidated through epigenetic regulation of claudin-5 expression: stress-resilient, but not stress-susceptible, animals exhibit permissive chromatin marks at the cldn5 locus together with low endothelial expression of the repressive transcription factor FOXO [110]. The resulting loss of barrier integrity facilitates the entry of peripheral pro-inflammatory cytokines into the brain, where they trigger microglial activation and subsequent emotional dysregulation [110]. Furthermore, VEGF has been identified as a critical mediator of stress-induced BBB disruption, with its overexpression increasing paracellular permeability and promoting the development of depressive-like behaviors [111]. Consistent with this concept, astrocytic cannabinoid receptor 1 (CB1) has been shown to promote stress resilience by attenuating stress-induced alterations in BBB integrity, highlighting the barrier as a dynamic component of the adaptive response of the brain to psychological stress [112]. In line with these preclinical findings, human genetic analyses implicate TNF-α signaling in the link between stress, barrier integrity, and depression [113].

Potential therapeutic mechanisms. These findings raise the possibility that stabilizing the BBB may itself have antidepressant potential, although the supporting evidence remains entirely preclinical. Edaravone, a free-radical scavenger, has been shown to ameliorate depressive- and anxiety-like behaviors in rodents through activation of the Sirt1/Nrf2/HO-1/Gpx4 pathway [114], and has also been shown to protect human brain endothelial cells against barrier disruption [115]. However, these effects of edaravone have not yet been experimentally linked in the context of depression. Similarly, in a mouse model of depression induced by chronic unpredictable stress, delivery of circDYM—a circular RNA analogue—via extracellular vesicles restored BBB integrity, suppressed neuroinflammation, and alleviated depressive-like behaviors [116]. To the best of our knowledge, no clinical trial has yet examined whether barrier-stabilizing agents can improve depressive symptoms. Collectively, these findings underscore that the BBB is a central integrator of systemic and central pathologies in MDD, moving the field beyond the traditional monoamine hypothesis toward a neurovascular framework.

### 4.2. Bipolar Disorder (BD)

BD is a severe, chronic psychiatric illness characterized by recurrent manic or hypomanic episodes that may alternate with depressive episodes [117,118]. Mania is marked by elevated or euphoric mood, increased activity and reduced need for sleep, rapid speech, impaired judgment, and, in some cases, increased sexual activity and aggressive behavior [117,118]. Emerging evidence positions “leaky brain” pathology not merely as a byproduct of mood episodes, but as a central mediator of long-term neural damage [42,119]. 

Evidence from patients. Clinical investigations based on the CSF/serum albumin ratio—a widely used indirect index of BBB permeability in humans—have consistently reported elevated ratios in BD patients, with the degree of leakage significantly correlating with illness duration and the number of manic episodes [64]. These findings are further supported by elevated peripheral levels of S100B. The latter is a calcium-binding protein primarily produced by astrocytes in the CNS; it exerts both neuroprotective and neurotoxic effects, depending on its levels [120]. It serves as a surrogate marker for astrocytic distress, acting as a damage-associated molecular pattern (DAMP) molecule and a biomarker for brain damage and neuroinflammation [121]. At high concentrations it signals neural distress and acute BBB breach [122]. Its interpretation, however, requires caution, as S100B is also produced peripherally including by adipocytes, a relevant confounder given the high prevalence of obesity among patients with BD [123].

Mechanisms of barrier breakdown. The molecular mechanisms underlying BBB breakdown in BD involve a complex interplay between systemic inflammation and the enzymatic degradation of the neurovascular unit. Chronic elevation of pro-inflammatory cytokines in BD stimulates the activity of MMPs, particularly MMP-9, which targets and degrades essential TJ proteins such as claudin-5 and occludin [42,124,125]. This structural breakdown is exacerbated by comorbid metabolic dysregulation. Specifically, insulin resistance and altered adipokine signaling, highly prevalent in BD, have been shown to directly impair endothelial function and reduce BBB integrity [126]. Insulin resistance is present in over half of patients with BD, and neuroimaging studies in living patients have shown that the severity of BBB leakage is proportional to illness severity and associated with insulin resistance, positioning this metabolic–vascular axis as a potential mechanism of neuroprogression [126]. These findings provide a mechanistic link between metabolic disturbances, BBB dysfunction, and the pathophysiological manifestations of BD [126]. Furthermore, BBB disruption in BD facilitates a self-perpetuating cycle in which peripheral oxidative stress markers, such as malondialdehyde, penetrate the CNS and overwhelm endogenous antioxidant defenses, including glutathione [45,119,127]. This shift toward a pro-oxidative state contributes to sustained neuroinflammatory signaling, which has been implicated in structural brain alterations, including white matter hyperintensities and cortical thinning, as well as the progressive cognitive decline observed in later stages of the disorder [119,128,129]. 

Potential therapeutic implications. Restoration of BBB integrity and neurovascular unit function may represent a key therapeutic target for mitigating neuroprogression and improving clinical outcomes in BD [119]. Unlike MDD, where barrier-directed strategies remain confined to preclinical models, preliminary human evidence exists in BD. A case report described amelioration of BBB dysfunction and clinical remission following treatment of insulin resistance in a patient with treatment-resistant bipolar depression [130]. Although based on a single patient, this observation raises the possibility that barrier compromise in BD may be reversible and that the metabolic axis may represent a modifiable point of intervention.

### 4.3. Schizophrenia

Schizophrenia is often regarded as one of the most severe psychiatric illnesses [131,132,133]. It primarily presents with psychotic symptoms (including positive symptoms such as delusions and hallucinations and negative symptoms such as blunted affect, anhedonia, social withdrawal, and reduced speech), cognitive impairment, severe functional disability, and a chronic course, and is associated with increased mortality [131,132,133]. 

Evidence from patients. CSF studies have reported increased BBB permeability in a subset of patients with schizophrenia. An individual patient data meta-analysis in recent-onset psychosis found CSF alterations to be associated with symptom severity [134], while inflammatory changes in CSF have likewise been described in paranoid schizophrenia [135]. Together, these findings suggest that barrier and immune involvement may be present from the early stages of illness rather than emerging only with chronicity. Moreover, these observations have been discussed in the context of the mechanisms underlying barrier disruption in the disorder [43]. More direct in vivo evidence comes from DCE-MRI, which has demonstrated increased thalamic permeability that correlates with symptom severity as assessed by the Positive and Negative Syndrome Scale [40]. On the other hand, a second study reported increased barrier leakage across several regions, without a corresponding association with symptom severity [38]. Beyond permeability itself, neuroimaging studies have identified reduced cerebral blood flow and altered endothelial integrity in the prefrontal cortex and temporal lobes, regions implicated in the cognitive and social deficits of the disorder [136,137,138]. At the tissue level, post-mortem studies have revealed significant downregulation of claudin-5, particularly in the hippocampus, consistent with a localized disruption of the paracellular barrier [50]. Supporting this observation, endothelial cells obtained from patient-derived organoids exhibit increased permeability and altered angiogenic patterns [139]. Molecular evidence of inflammation-driven angiogenesis and endothelial alterations that permit macrophage diapedesis has also been described in the midbrain, with these changes being most pronounced in a high-inflammation subgroup [71] (see Section 3.1).

Mechanisms of barrier breakdown. Recent research highlights the critical role of pericyte dysfunction; these cells, which are essential for maintaining BBB stability, show reduced pericyte coverage of brain capillaries and altered signaling in schizophrenia, potentially mediated by dysregulated TGF-β and PDGF pathways [140]. This pericyte loss not only increases barrier permeability but also impairs the clearance of neurotoxic metabolites, further exacerbating oxidative stress and microglial activation [141]. The “leaky” BBB in schizophrenia acts as a gateway for peripheral pro-inflammatory mediators to infiltrate the brain, thereby sustaining a state of chronic neuroinflammation. Elevated levels of MMP-9 and pro-inflammatory cytokines such as IL-6 have been shown to correlate with both BBB disruption and the severity of positive and negative symptoms among patients with schizophrenia [124,125]. 

Potential therapeutic implications. The neurovascular dysfunction presumed to occur in patients with schizophrenia may impair the transport of antipsychotic medications, potentially contributing to the clinical challenge of treatment resistance [5]. This is particularly relevant considering the role of efflux transporters, such as P-glycoprotein, in restricting the central availability of medications. Whether barrier disruption is more pronounced in patients with treatment-resistant illness and contributes causally to poor treatment response remains to be established. Therefore, therapeutic strategies aimed at stabilizing pericyte–endothelial interactions and restoring TJ integrity offer a novel paradigm for mitigating the progressive neurobiological changes associated with schizophrenia. However, as in MDD, these strategies have not yet been evaluated clinically.

Collectively, these findings underscore that MDD, BD, and schizophrenia transcend the traditional neurotransmitter-centric models, involving systemic neurovascular components that alter brain homeostasis. Rather than a secondary phenomenon, BBB dysfunction emerges as a critical integrator of peripheral–central crosstalk, driving disease progression, therapeutic resistance, and the neuroprogressive nature of these disorders.

## 5. Therapeutic Implications

Restoration of BBB integrity has emerged as a promising therapeutic strategy in neuropsychiatric disorders, as the BBB represents a repairable and clinically relevant target whose stabilization can mitigate neuroinflammation, oxidative stress, vascular dysfunction, and downstream neuroimmune and neuronal disturbances [136,137]. Recent studies identified multiple mechanistic entry points for therapeutic intervention, including anti-inflammatory and antioxidant modulation, endothelial stabilization, metabolic correction, and novel BBB-focused drug delivery approaches [136,142,143].

### 5.1. Anti-Inflammatory and Antioxidant Strategies

Given the central role of neuroinflammation and oxidative stress in BBB dysfunction, pharmacologic strategies targeting these pathways hold considerable promise [45,76,144]. Agents such as minocycline, N-acetylcysteine (NAC), and curcumin derivatives have demonstrated efficacy in preclinical and early clinical studies by attenuating cytokine production, reducing ROS, and upregulating TJ [145,146]. For instance, NAC elevates endothelial glutathione levels and mitigates oxidative injury in both rodent and human BBB models [147,148,149,150]. Similarly, polyphenols (e.g., resveratrol, quercetin) exert antioxidant and anti-inflammatory effects via NF-κB inhibition and Nrf2 pathway activation, which collectively preserve endothelial function and barrier integrity [151,152,153,154].

A related avenue involves the gasotransmitters nitric oxide (NO) and hydrogen sulfide (H2S), which regulate endothelial tone and barrier permeability and may therefore represent mechanistic link between redox status and BBB integrity. Notably, these two molecules are functionally coupled, as H2S stimulates NO release from cerebrovascular endothelial cells [155]. In schizophrenia, dysregulated NO signaling and excessive NO production under conditions of oxidative stress and inflammation have been implicated in neuronal and glial damage [156]. Nonetheless, sodium nitroprusside, a NO donor and potent vasodilator, has been investigated as a treatment for schizophrenia [157], highlighting the complex, concentration-dependent nature of NO signaling. Regarding H2S, some patients with schizophrenia have been shown to have reduced plasma levels, with lower levels being associated with greater severity of negative symptoms [158]. Of note, experimental studies have reported that H2S preserves barrier integrity by inhibiting MMP-9-mediated TJ degradation and upregulating claudin-5, occludin, and ZO-1 [159,160]. Evidence in mood disorders remains limited, and whether modulation of gasotransmitter signaling alters BBB permeability in psychiatric populations has not been directly assessed.

Furthermore, various anti-inflammatory treatments have been investigated as potential therapeutic strategies for mental disorders, including MDD, BD, and schizophrenia [161,162,163,164,165,166,167,168,169,170]. Nonetheless, it should be noted that the evidence regarding the therapeutic efficacy of anti-inflammatory agents in the treatment of these disorders remains inconclusive, as conflicting findings have also been reported [171,172,173,174,175,176]. Biological therapies targeting pro-inflammatory cytokines (e.g., TNF-α or IL-1β inhibitors) are also being investigated for their potential to indirectly reduce BBB permeability and neuroinflammatory signaling [103,177,178]. Although current evidence remains preliminary, these interventions highlight the translational potential of systemic anti-inflammatory therapy in stabilizing neurovascular health.

### 5.2. Therapeutic Agents Modulating Endothelial Function and TJs

Endothelial stabilization represents another key therapeutic frontier. Endothelial NO synthase (eNOS) is an enzyme involved in NO synthesis and the regulation of vascular tone. Under physiological conditions, eNOS generates NO from L-arginine using tetrahydrobiopterin (BH4) as a cofactor, thereby supporting vasodilation, limiting leukocyte adhesion, and maintaining barrier integrity [179,180,181,182]. eNOS has a dual role, serving as a source of protective NO under physiological conditions; however, when dysfunctional, it contributes to oxidative injury through the generation of reactive oxygen species. When BH4 availability becomes limiting, as occurs under oxidative stress, eNOS becomes enzymatically “uncoupled” and generates superoxide instead of NO [183,184]. This process can further exacerbate eNOS uncoupling, as superoxide oxidizes BH4 to BH2 while scavenging residual NO to form peroxynitrite, a potent oxidant that nitrates junctional and matrix proteins. These findings make eNOS a potential mechanistic link between vascular dysfunction, oxidative stress, and BBB dysfunction across various neurological diseases, including psychiatric disorders. Consistently, statin medications, beyond their lipid-lowering properties, have been shown to enhance eNOS activity and reduce oxidative stress, thereby improving endothelial resilience and preserving TJ integrity [185,186]. Angiotensin receptor blockers (ARBs), such as losartan and candesartan, exhibit similar vasculo-protective and anti-inflammatory effects that extend to the cerebral vasculature [187,188].

In the context of mental illness, specifically schizophrenia, reduced eNOS activity and eNOS uncoupling have been proposed as a mechanistic link between neuroinflammation, oxidative stress, and BBB hyperpermeability, integrating peroxynitrite formation, MMP activation, and VEGF signaling within a single neurovascular framework [189]. Furthermore, in mice, chronic unpredictable mild stress and chronic social defeat stress produced cerebral microvascular dysfunction, which preceded and predicted the emergence of depressive-like behavior [190]. On the other hand, eNOS-deficient (eNOS+/−) mice—a classic model of endothelial injury—developed depressive-like behavior even in the absence of stress exposure, with these behavioral changes emerging approximately two months after the onset of microvascular dysfunction [190]. This pattern suggests that endothelial NO deficiency precedes the behavioral phenotype rather than resulting from it. Consistent with these findings, reduced plasma NO metabolite levels have been reported in patients with depression [191], while antidepressant treatment has been shown to partially reverse these changes [192]. Whether interventions aimed at restoring eNOS coupling—such as BH4 supplementation or statin therapy—can normalize BBB permeability in psychiatric populations remains untested.

Recent studies have identified small molecules and peptides capable of directly modulating TJ proteins, offering a promising avenue for restoring barrier integrity [193,194]. A key pathway in this context is Wnt/β-catenin signaling, which is essential for CNS angiogenesis and the maintenance of endothelial TJ expression [195,196]. Interestingly, classic psychotropic medications, most notably lithium, are potent activators of the Wnt/β-catenin pathway through the inhibition of glycogen synthase kinase-3beta (GSK-3β) [197,198]. Emerging evidence suggests that the therapeutic efficacy of lithium in BD may be partially mediated by its ability to stabilize the BBB, increase claudin-5 expression, and reduce paracellular permeability [119,199,200]. Similarly, pharmacological activation of the Shh pathway has been shown to reinforce the barrier by upregulating occludin and junctional adhesion molecules in inflammatory models [201].

The role of GR agonists, such as dexamethasone, in modulating BBB integrity is also critical [101]. While traditionally used to treat acute CNS edema, GR agonists have been shown to directly enhance the transcription of TJ proteins, specifically occludin and claudin-5, via glucocorticoid response elements (GRE) in their promoter regions [202,203,204,205]. However, the dosing context is vital; while acute activation reinforces the barrier, chronic GC exposure, as seen in chronic stress or prolonged steroid therapy, can lead to a paradoxical downregulation of TJ proteins and increased BBB vulnerability [206]. Thus, understanding the temporal effects of both classical psychotropics and hormonal modulators is essential for translating neurovascular-targeted strategies into clinical practice.

### 5.3. Novel Therapeutic Approaches

Emerging nanotechnology and biotechnology platforms are revolutionizing how therapies interact with the BBB. Nanocarriers (liposomes, polymeric nanoparticles, exosomes) can be engineered to cross the BBB selectively via receptor-mediated transcytosis or adsorptive transport, enabling precise drug delivery to neural targets while minimizing systemic toxicity [207,208,209,210,211].

While pathological BBB hyperpermeability contributes to neuroinflammation and neural dysfunction in neuropsychiatric disorders, controlled and targeted modulation of the barrier can be therapeutically beneficial. In this context, the goal shifts from preventing maladaptive, uncontrolled leakage to enabling precise, regulated transport of therapeutic agents across the BBB. Thus, strategies aimed at restoring BBB integrity coexist with approaches designed to safely and selectively facilitate drug delivery into the CNS.

Additionally, biological vectors such as engineered exosomes and viral nanoparticles are being explored for gene and RNA delivery aimed at restoring endothelial stability, modulating inflammatory gene expression, or correcting transporter dysfunction [212,213,214]. Techniques such as focused ultrasound (FUS) combined with microbubbles offer a controlled, reversible method to transiently open the BBB, allowing targeted therapeutic entry while maintaining long-term safety [215,216].

BBB-targeted delivery is currently considered essential for optimizing central drug bioavailability, reducing peripheral side effects, and achieving sustained neurochemical modulation in disorders like depression and schizophrenia [43,48,65,217,218].

### 5.4. Lifestyle and Systemic Factors Affect BBB Integrity

Beyond pharmacologic interventions, modifiable lifestyle and systemic factors play a significant role in BBB maintenance [143,219]. Regular aerobic exercise enhances cerebral blood flow, upregulates endothelial NO signaling, and promotes antioxidant capacity in the NVU. Dietary interventions—particularly those rich in omega-3 fatty acids, flavonoids, and antioxidants—have been associated with improved BBB integrity and reduced systemic inflammation [220,221,222,223]. Moreover, management of metabolic comorbidities such as insulin resistance, hypertension, and dyslipidemia indirectly support BBB integrity by mitigating endothelial stress and microvascular damage [126,224]. Stress reduction and sleep regulation further reduce GC- and cytokine-driven endothelial activation, highlighting the potential of integrated behavioral and medical strategies to preserve neurovascular function in psychiatric populations [28,225].

Following are three tables (Table 1, Table 2 and Table 3) presenting the main studies addressing the relationship between BBB dysfunction and the discussed psychiatric disorders. Only original studies reporting results directly related to BBB dysfunction are included in the tables; review articles cited in the text are excluded.

**Table 1:** Key studies linking blood–brain barrier dysfunction to MDD.

Ref.	Study Type	Population/Model	BBB Marker or Mechanism	Main Findings
[73]	Preclinical (male mice) + human post-mortem tissue	Chronic social defeat stress in mice; NAc tissue from donors who died during a depressive episode	Claudin-5 expression; peripheral IL-6 entry	Claudin-5 was reduced in NAc of stress-susceptible but not resilient mice; viral knockdown of claudin-5 alone was sufficient to induce depression-like behavior after stress, which was reversed by chronic antidepressant treatment. *CLDN5* was also reduced in human NAc
[74]	Preclinical (female mice) + human post-mortem tissue	Chronic stress in female mice; human brain tissue	Claudin-5 in prefrontal cortex; vascular integrity	Stress-induced claudin-5 loss in PFC was accompanied by anxiety-like and depression-like behavior; parallel reduction in human depressed tissue
[109]	Clinical (human subjects)	Healthy controls and patients with MDD	Plasma claudin-5	Elevated plasma claudin-5 were associated with increased peripheral inflammation and BBB breakdown
[110]	Preclinical (male mice)	Chronic social defeat stress in mice	Epigenetic regulation of claudin-5; TNF-α infiltration	TNF-α/NF-κB signaling mediates stress susceptibility; restoration of stress-induced claudin-5 deficiency in the NAc promotes resilience
[113]	Clinical/genetic	Human cohort	TNF-α signaling and BBB integrity	TNF-α implicated as a mediator between stress and barrier integrity in depression
[111]	Preclinical (male mice)	Stress-exposed mice	VEGF-mediated BBB permeability	VEGF over-expression increases paracellular BBB permeability and depressive-like behavior
[112]	Preclinical (mice) + human post-mortem tissue	Chronic stress in mice; brain tissue from males with MDD	Astrocytic CB1	CB1 signaling mitigates stress-induced BBB dysfunction and promotes resilience; loss of CB1 gene (*Cnr1*) in NAc of MDD patients
[114]	Preclinical (male mice)	Mouse model of depression	Sirt1/Nrf2/HO-1/Gpx4 pathway	Edaravone ameliorates depressive-like and anxiety-like behavior
[115]	In vitro (brain endothelial cell line)	Human brain endothelial cells	Barrier damage induced by methylglyoxal	Edaravone protects against endothelial barrier damage
[116]	Preclinical (mice)	Chronic unpredictable stress model in mice	circDYM delivered via extracellular vesicles	circDYM inhibits microglial activation and suppresses neuroinflammation; cirDYM restores BBB integrity

Abbreviations: BBB, blood–brain barrier; CB1, cannabinoid receptor 1; circDYM, circular RNA DYM; *Cnr1*, cannabinoid receptor 1 gene; *CLDN5*, claudin-5 gene; Gpx4, glutathione peroxidase 4; HO-1, heme oxygenase 1; IL-6, interleukin 6; MDD, major depressive disorder; NAc, nucleus accumbens; NF-κB, nuclear factor κB; Nrf2, nuclear factor erythroid 2-related factor 2; PFC, prefrontal cortex; Sirt1, sirtuin 1; TNF-α, tumor necrosis factor-α; VEGF, vascular endothelial growth factor.

**Table 2:** Key studies linking blood–brain barrier dysfunction to BD.

Ref.	Study Type	Population/Model	BBB Marker or Mechanism	Main Findings
[124]	Clinical (human subjects, males and females)	Healthy controls, and patients with ADHD, BD, or MDD	MMP-9; CRP; IL-6	MMP-9 and cytokine levels differed by diagnosis and by severity of emotional dysregulation
[125]	Clinical (human subjects, males and females)	Healthy controls, and patients with BD or schizophrenia	Plasma MMP-9	MMP-9 elevated in both disorders; influenced by modifiable factors
[126]	Clinical (human subjects, males and females)	Healthy controls and patients with BD	BBB leakage (DCE-MRI); insulin resistance	BBB leakage was proportional to illness severity and associated with insulin resistance
[130]	Clinical (case report)	A patient with treatment-resistant BD	BBB leakage on imaging; insulin resistance	Amelioration of insulin resistance was accompanied by reversal of BBB dysfunction and clinical remission

Abbreviations: ADHD, attention-deficit/hyperactivity disorder; BBB, blood–brain barrier; BD, bipolar disorder; CRP, C-reactive protein; DCE-MRI, dynamic contrast-enhanced magnetic resonance imaging; IL-6, interleukin 6; MDD, major depressive disorder; MMP-9, matrix metalloproteinase 9.

**Table 3:** Key studies linking blood–brain barrier dysfunction to schizophrenia.

Ref.	Study Type	Population/Model	BBB Marker or Mechanism	Main Findings
[38]	Clinical (human subjects, males and females)	Healthy controls and patients with schizophrenia	Regional BBB leakage (DCE-MRI)	Patients with schizophrenia had higher leakage across several regions, without association with symptom severity
[40]	Clinical (human subjects, males and females)	Healthy controls and patients with schizophrenia	Thalamic permeability (DCE-MRI)	Increased thalamic permeability correlated with severity of symptoms (PANSS) and alterations in brain volume
[50]	Human post-mortem tissue (males and females)	Brain tissue (seven regions) from control subjects, and patients with schizophrenia, MDD, and BD	Claudin-5, claudin-12, ZO-1 (qRT-PCR and immunohistochemistry)	Expression of claudin-5 was reduced in the hippocampus of patients with schizophrenia and MDD; transcript levels of TJ mRNA of claudin-5, claudin-12 and ZO-1 were correlated with illness duration and age-of-onset
[64]	Clinical (human subjects, males and females)	Healthy controls and patients with psychotic disorders	CSF/serum albumin ratio; CSF inflammatory markers	Increased albumin ratio (=increased BBB permeability) and neuroinflammation in BD patients; severity of BBB leakage was associated with illness duration and number of manic episodes
[71]	Human post-mortem tissue (males and females)	Midbrain tissue from control subjects, and patients with schizophrenia and BD	Angiogenesis markers; macrophage diapedesis	Increased inflammation-associated angiogenesis seemed to correlate with BBB dysfunction in subgroups of patients with schizophrenia and BD
[135]	Clinical (human subjects, males and females)	Healthy controls and patients with paranoid schizophrenia	CSF inflammatory biomarkers	Patients with schizophrenia had higher CSF inflammatory markers; increased inflammation was associated with illness duration and higher frequency of psychotic episodes
[139]	In vitro (cells from males and females)	Pluripotent stem cells derived from schizophrenia patients and control subjects	Permeability; angiogenesis patterns	Schizophrenia-derived endothelial cells show altered angiogenesis and higher permeability

Abbreviations: BBB, blood–brain barrier; BD, bipolar disorder; CSF, cerebrospinal fluid; DCE-MRI, dynamic contrast-enhanced magnetic resonance imaging; MDD, major depressive disorder; mRNA, messenger RNA; PANSS, Positive and Negative Syndrome Scale; qRT-PCR, quantitative reverse transcription polymerase chain reaction; TJ, tight junction; ZO-1, zonula occludens 1.

## 6. Challenges and Knowledge Gaps

Despite remarkable progress in delineating the role of the BBB in psychiatric disorders, substantial methodological and conceptual challenges continue to limit the field’s ability to draw causal inferences and translate preclinical discoveries into effective human therapies [226,227]. Current understanding of BBB dysfunction is largely derived from animal and postmortem studies, which—while invaluable—often fail to capture the temporal dynamics, regional heterogeneity, and subtle permeability alterations characteristic of psychiatric illness [38,47,228,229]. Traditional experimental approaches frequently rely on static measurements of permeability markers or TJ protein expression, offering only a limited snapshot of a highly dynamic interface [193]. Moreover, in vitro BBB models, although increasingly sophisticated, still struggle to reproduce the complex three-dimensional architecture, shear stress, and multicellular interactions of the NVU that define in vivo physiology [230,231]. Even advanced organ-on-chip and induced pluripotent stem cell (iPSC)-derived systems exhibit variability in endothelial phenotype and transporter expression, complicating cross-study comparisons and reproducibility [232].

A critical translational obstacle arises from interspecies differences between rodent and human BBB biology [233]. Rodent endothelial cells differ in transporter density, immune responsiveness, and junctional composition, which affects permeability and medication kinetics [234,235]. Consequently, therapeutic candidates showing barrier-stabilizing effects in animals occasionally yield inconsistent outcomes in humans [236]. Yet, no animal model fully recapitulates human diseases, but, also, no individual patient’s condition fully recapitulates another patient’s condition. Conceivably, the closer to human the species is and the closer the pathogenesis is to the relevant human condition, the greater the confidence in the translatability of the results [234]. Psychiatric disorders pose an additional complication: they evolve over years and display fluctuating systemic influences, such as stress hormones and metabolic alterations that are difficult to mimic in acute animal paradigms [237,238]. Furthermore, psychiatric phenotypes themselves lack direct analogues in rodents, making it challenging to link molecular barrier changes to cognitive or affective symptoms with translational validity [239,240]. These limitations underscore the need for cross-species frameworks that integrate cellular, molecular, and behavioral endpoints, as well as the development of standardized biomarkers that can be assessed consistently across experimental platforms.

Equally important is the current fragmentation of methodological approaches across molecular, imaging, and clinical research. Studies often examine isolated aspects of BBB dysfunction, such as cytokine levels, MRI permeability indices, or transcriptomic signatures, without integrating these data into unified models of neurovascular pathology [47]. The field now requires multi-omics approaches that combine transcriptomic, proteomic, metabolomic, and epigenomic data to reveal the regulatory networks linking inflammation, oxidative stress, and endothelial signaling [47,241]. When aligned with longitudinal neuroimaging modalities, particularly DCE-MRI, diffusion-weighted imaging, and novel PET tracers, such datasets could enable temporal mapping of BBB alterations relative to symptom trajectories, treatment response, and disease progression [242,243,244]. However, implementing these integrative frameworks presents logistical and computational challenges, including data harmonization, cost, and the need for interdisciplinary expertise bridging psychiatry, vascular biology, and systems neuroscience [241,242].

Ultimately, overcoming these methodological and translational barriers will require standardized protocols, shared databases, and collaborative consortia capable of correlating molecular and imaging biomarkers with clinical phenotypes across large, diverse cohorts [245,246,247]. Only through such coordinated efforts can the field move from associative evidence toward a mechanistic and predictive understanding of BBB dysfunction in psychiatric disorders—an essential step toward developing targeted, precision-based interventions.

## 7. Future Directions

The growing recognition of the BBB as a dynamic and responsive interface in psychiatric disorders has opened a new frontier for translational research [219,248]. Future work must advance beyond descriptive associations to establish mechanistic, predictive, and clinically actionable frameworks that position the BBB at the center of diagnostic and therapeutic innovation [249]. Three interrelated directions—biomarker development, therapeutic targeting, and neurovascular model integration—are particularly crucial for transforming current understanding into precision medicine strategies [42,119,250]. One of the most promising avenues lies in the development of biomarker-based approaches capable of detecting subtle BBB alterations and linking them to specific clinical phenotypes. Advances in imaging modalities, such as DCE-MRI, diffusion-weighted imaging, and PET tracers for endothelial and glial activation, enable in vivo quantification of BBB permeability and inflammation with unprecedented spatial and temporal resolution [228]. When combined with CSF, blood and saliva biomarkers, such as the albumin quotient (QAlb) [251], S100B [121,252,253,254], glial fibrillary acidic protein [121,254], MMPs [255], and endothelial-derived extracellular vesicles [256,257], these tools could form a multimodal biomarker panel capable of stratifying patients according to neurovascular dysfunction. Integrating such markers into clinical trials would facilitate earlier detection of BBB impairment, monitoring of treatment response, and identification of individuals most likely to benefit from barrier stabilization.

Restoring endothelial and glial homeostasis offers the potential to not only reduce neuroinflammation and oxidative stress but also indirectly normalize neurotransmission and synaptic function [142]. Future therapeutic strategies may include small molecules that enhance TJ integrity, modulators of Wnt/β-catenin or Shh signaling, or agents targeting pericyte–endothelial crosstalk to reinforce vascular stability [142,195]. In addition, metabolic modulators and antioxidant compounds could be deployed in combination with psychotropic drugs to address both neural and vascular dysfunction. Clinical translation will benefit from the incorporation of BBB endpoints, such as imaging-based permeability indices and circulating vascular biomarkers, into psychiatric treatment trials, allowing assessment of whether restoring barrier integrity correlates with symptom improvement and functional recovery [258].

Up to this point, the discussion has addressed neurovascular modeling and BBB-focused strategies in a broad, cross-disciplinary context. However, these principles gain particular significance when considered within psychiatric research, where BBB dysfunction is increasingly viewed not merely as a secondary correlate, but as a potential contributor to symptom emergence, treatment response, and disease progression. Against this backdrop, the following section narrows the focus to the psychiatric domain. 

The integration of neurovascular models into psychiatric research will be essential for capturing the bidirectional interactions between systemic physiology, the BBB, and neural circuitry. Advanced in vitro models, including iPSC-derived NVU systems, organ-on-chip technologies, and 3D microfluidic platforms, now allow precise manipulation of human endothelial, astrocytic, and pericytic components under controlled conditions [259,260]. These systems, coupled with multi-omics profiling and computational modeling, can elucidate causal pathways linking molecular insults to barrier dysfunction and neural outcomes [261]. In parallel, system-level models that merge vascular biology with neuroimaging and behavioral data will help clarify how peripheral inflammation, stress hormones, and metabolic changes translate into psychiatric symptoms. Such integrative frameworks could ultimately yield predictive models of disease progression, bridging the gap between molecular mechanisms and clinical manifestations.

By embracing the BBB as both a biomarker source and a therapeutic target, the field stands to develop more holistic and personalized strategies for understanding and treating complex mental illnesses. Achieving this vision will depend on sustained interdisciplinary collaboration among neuroscientists, psychiatrists, immunologists, and vascular biologists, supported by longitudinal cohort studies and open data-sharing initiatives. Only through such coordinated efforts can the full diagnostic and therapeutic potential of the BBB be realized, transforming our approach to psychiatric disorders in the decades ahead.

## 8. Conclusions

The evidence discussed in this review underscores a transformative understanding of the BBB as an active player in psychiatric pathophysiology. The reviewed studies provide strong evidence suggesting that the loss of claudin-5 is associated with BBB dysfunction in psychiatric disorders. For instance, its expression is reduced in post-mortem brains of patients with MDD and schizophrenia, while in rodents, its knockdown in the brain is sufficient to induce depressive-like behavior, which is reversed by antidepressant treatment. In BD, the metabolic–vascular axis is the best-characterized, with in vivo imaging studies linking insulin resistance to BBB leakage and severity of illness. Evidence for oxidative and mitochondrial contributions is robust in peripheral tissues but only partly disorder-specific, whereas evidence for gasotransmitter- and endothelium-targeted mechanisms remains largely preclinical. Notably, BBB dysfunction appears to be a shared feature across all three disorders, whereas its regional distribution and molecular drivers differ. To date, no BBB-directed therapy has been evaluated in a controlled clinical trial. By positioning the BBB as a biomarker source, therapeutic target, and mechanistic bridge between body and brain, future research can foster more predictive, personalized, and biologically grounded approaches to mental health treatment, transforming how psychiatric disorders are studied, diagnosed, and managed.

## Data Availability

No new data were created or analyzed in this study. Data sharing is not applicable to this article.

## Abbreviations

The following abbreviations are used in this manuscript:

Ang1	Angiopoietin 1
Ang2	Angiopoietin 2
Ang/Tie	Angiopoietin/Tie signaling pathway
ARBs	Angiotensin receptor blockers
BBB	Blood–brain barrier
BD	Bipolar disorder
BH4/BH2	Tetrahydrobiopterin/Dihydrobiopterin
CB1	Cannabinoid receptor 1
*CLDN5*	Claudin-5 gene
CNS	Central nervous system
CSF	Cerebrospinal fluid
DAMP	Damage-associated molecular pattern
DCE-MRI	Dynamic contrast-enhanced magnetic resonance imaging
ECF	Extracellular fluid
ECM	Extracellular matrix
eNOS	Endothelial nitric oxide synthase
EZH2	Enhancer of zeste homolog 2
FUS	Focused ultrasound
GCs	Glucocorticoids
GDNF	Glial-derived neurotrophic factor
GR	Glucocorticoid receptor
GRE	Glucocorticoid response element
GSK-3β	Glycogen synthase kinase-3 beta
HPA axis	Hypothalamic–pituitary–adrenal axis
IFN-γ	Interferon gamma
IL	Interleukin
IL-1β	Interleukin 1 beta
IL-6	Interleukin 6
iPSC	Induced pluripotent stem cells
JAMs	Junctional adhesion molecules
MAPK	Mitogen-activated protein kinase
MDD	Major depressive disorder
MMPs	Matrix metalloproteinases
MRI	Magnetic resonance imaging
MRPs	Multidrug resistance-associated proteins
NAC	N-acetylcysteine
NF-κB	Nuclear factor kappa B
NO	Nitric oxide
NVU	Neurovascular unit
PANSS	Positive and Negative Syndrome Scale
P-gp	P-glycoprotein
PDGF-BB	Platelet-derived growth factor BB
PDGFR-β	Platelet-derived growth factor receptor beta
PET	Positron emission tomography
QAlb	Albumin quotient
ROS	Reactive oxygen species
S100B	S100 calcium-binding protein B
SANRA	Scale for the Assessment of Narrative Review Articles
Shh	Sonic Hedgehog
TGF-β	Transforming growth factor beta
Tie2	Tyrosine kinase with immunoglobulin-like and EGF-like domains 2
TJ/TJs	Tight junction/Tight junctions
TNF-α	Tumor necrosis factor alpha
VEGF	Vascular endothelial growth factor
ZO-1/2/3	Zonula occludens proteins 1/2/3

## References

[1] Ding J., Jiang Y., Jiang N., Xing S., Ge F., Ma P., Tang Q., Miao H., Zhou J., Fang Y. (2025). Bridging the Gap: Unlocking the Potential of Emerging Drug Therapies for Brain Metastasis. Brain.

[2] Armulik A., Genové G., Mäe M., Nisancioglu M.H., Wallgard E., Niaudet C., He L., Norlin J., Lindblom P., Strittmatter K. (2010). Pericytes Regulate the Blood-Brain Barrier. Nature.

[3] Obermeier B., Daneman R., Ransohoff R.M. (2013). Development, Maintenance and Disruption of the Blood-Brain Barrier. Nat. Med..

[4] Daneman R., Prat A. (2015). The Blood–Brain Barrier. Cold Spring Harb. Perspect. Biol..

[5] Archie S.R., Al Shoyaib A., Cucullo L. (2021). Blood-Brain Barrier Dysfunction in CNS Disorders and Putative Therapeutic Targets: An Overview. Pharmaceutics.

[6] ElAli A., Thériault P., Rivest S. (2014). The Role of Pericytes in Neurovascular Unit Remodeling in Brain Disorders. Int. J. Mol. Sci..

[7] Dotiwala A.K., McCausland C., Samra N.S. (2023). Anatomy, Head and Neck: Blood Brain Barrier.

[8] Alahmari A. (2021). Blood-Brain Barrier Overview: Structural and Functional Correlation. Neural Plast..

[9] Dithmer S., Blasig I.E., Fraser P.A., Qin Z., Haseloff R.F. (2024). The Basic Requirement of Tight Junction Proteins in Blood-Brain Barrier Function and Their Role in Pathologies. Int. J. Mol. Sci..

[10] Benarroch E. (2023). What Are the Roles of Pericytes in the Neurovascular Unit and Its Disorders?. Neurology.

[11] Kugler E.C., Greenwood J., MacDonald R.B. (2021). The “Neuro-Glial-Vascular” Unit: The Role of Glia in Neurovascular Unit Formation and Dysfunction. Front. Cell Dev. Biol..

[12] Ronaldson P.T., Davis T.P. (2020). Regulation of Blood–Brain Barrier Integrity by Microglia in Health and Disease: A Therapeutic Opportunity. J. Cereb. Blood Flow Metab..

[13] Gomez-Zepeda D., Taghi M., Scherrmann J.M., Decleves X., Menet M.C. (2019). ABC Transporters at the Blood–Brain Interfaces, Their Study Models, and Drug Delivery Implications in Gliomas. Pharmaceutics.

[14] Ahmed Juvale I.I., Abdul Hamid A.A., Abd Halim K.B., Che Has A.T. (2022). P-Glycoprotein: New Insights into Structure, Physiological Function, Regulation and Alterations in Disease. Heliyon.

[15] Kaya M., Ahishali B. (2020). Basic Physiology of the Blood-Brain Barrier in Health and Disease: A Brief Overview. Tissue Barriers.

[16] Durairaj P., Liu Z.L. (2025). Brain Cytochrome P450: Navigating Neurological Health and Metabolic Regulation. J. Xenobiot..

[17] Zhou L., Wu Q., Jiang L., Rao J., Gao J., Zhao F., Wang X. (2025). Role of the Microbiota in Inflammation-Related Related Psychiatric Disorders. Front. Immunol..

[18] Gruenbaum B.F., Zlotnik A., Fleidervish I., Frenkel A., Boyko M. (2022). Glutamate Neurotoxicity and Destruction of the Blood-Brain Barrier: Key Pathways for the Development of Neuropsychiatric Consequences of TBI and Their Potential Treatment Strategies. Int. J. Mol. Sci..

[19] Hawkins R.A., Viña J.R. (2016). How Glutamate Is Managed by the Blood-Brain Barrier. Biology.

[20] Hussain B., Fang C., Huang X., Feng Z., Yao Y., Wang Y., Chang J. (2022). Endothelial β-Catenin Deficiency Causes Blood-Brain Barrier Breakdown via Enhancing the Paracellular and Transcellular Permeability. Front. Mol. Neurosci..

[21] Hill S.A., Fu M., Garcia A.D.R. (2020). Sonic Hedgehog Signaling in Astrocytes. Cell. Mol. Life Sci..

[22] Mannan A., Dhimann S., Gargn N., Singh T.G. (2023). Pharmacological Modulation of Sonic Hedgehog Signaling Pathways in Angiogenesis: A Mechanistic Perspective. Dev. Biol..

[23] Michinaga S., Hishinuma S., Koyama Y. (2024). Roles of Astrocytic Sonic Hedgehog Production and Its Signal for Regulation of the Blood-Brain Barrier Permeability. Vitam. Horm..

[24] Sha L., Zhao Y., Li S., Wei D., Tao Y., Wang Y. (2024). Insights to Ang/Tie Signaling Pathway: Another Rosy Dawn for Treating Retinal and Choroidal Vascular Diseases. J. Transl. Med..

[25] Chaudhary V., Mar F., Amador M.J., Chang A., Gibson K., Joussen A.M., Kim J.E., Lee J., Margaron P., Saffar I. (2024). Emerging Clinical Evidence of a Dual Role for Ang-2 and VEGF-A Blockade with Faricimab in Retinal Diseases. Graefe’s Arch. Clin. Exp. Ophthalmol..

[26] Akwii R.G., Mikelis C.M. (2021). Targeting the Angiopoietin/Tie Pathway: Prospects for Treatment of Retinal and Respiratory Disorders. Drugs.

[27] Lee C., Kim M.J., Kumar A., Lee H.W., Yang Y., Kim Y. (2025). Vascular Endothelial Growth Factor Signaling in Health and Disease: From Molecular Mechanisms to Therapeutic Perspectives. Signal Transduct. Target. Ther..

[28] Zhou M.Y., Xie F., Zhao Y., Wang X., Sun Z.W., Qian L.J. (2025). Stress-Induced Regional Characteristics of the Blood-Brain Barrier. Brain Res. Bull..

[29] Selim M.S., Matani B.R., Henry-Ojo H.O., Narayanan S.P., Somanath P.R. (2025). Claudin 5 Across the Vascular Landscape: From Blood–Tissue Barrier Regulation to Disease Mechanisms. Cells.

[30] Welcome M.O., Mastorakis N.E. (2020). Stress-Induced Blood Brain Barrier Disruption: Molecular Mechanisms and Signaling Pathways. Pharmacol. Res..

[31] Lee S., Kang B.M., Kim J.H., Min J., Kim H.S., Ryu H., Park H., Bae S., Oh D., Choi M. (2018). Real-Time in Vivo Two-Photon Imaging Study Reveals Decreased Cerebro-Vascular Volume and Increased Blood-Brain Barrier Permeability in Chronically Stressed Mice. Sci. Rep..

[32] Galea I. (2021). The Blood–Brain Barrier in Systemic Infection and Inflammation. Cell. Mol. Immunol..

[33] Divecha Y.A., Rampes S., Tromp S., Boyanova S.T., Fleckney A., Fidanboylu M., Thomas S.A. (2025). The Microcirculation, the Blood-Brain Barrier, and the Neurovascular Unit in Health and Alzheimer Disease: The Aberrant Pericyte Is a Central Player. Pharmacol. Rev..

[34] Li G., Gao J., Ding P., Gao Y. (2024). The Role of Endothelial Cell–Pericyte Interactions in Vascularization and Diseases. J. Adv. Res..

[35] Santomauro D.F., Miller P.A., Shadid J., Wulf Hanson S., Vo A., Roy D.J., Hagins H., Mantilla Herrera A.M., Scott J.G., Erskine H.E. (2026). Updated Trends in the Global Prevalence and Burden of Mental Disorders, 1990–2023: A Systematic Analysis for the Global Burden of Disease Study 2023. Lancet.

[36] Narrow W.E., Rae D.S., Robins L.N., Regier D.A. (2002). Revised Prevalence Estimates of Mental Disorders in the United States: Using a Clinical Significance Criterion to Reconcile 2 Surveys’ Estimates. Arch. Gen. Psychiatry.

[37] Ringeisen H., Edlund M., Guyer H., Dever J., Carpenter L., Olfson M., First M., Geiger P., Liao D., Peytchev A. (2025). Prevalence of Past-Year Mental and Substance Use Disorders, 2021–2022. Psychiatr. Serv..

[38] Moussiopoulou J., Yakimov V., Roell L., Rauchmann B.S., Toth H., Melcher J., Jäger I., Lutz I., Kallweit M.S., Papazov B. (2025). Higher Blood–Brain Barrier Leakage in Schizophrenia-Spectrum Disorders: A Comparative Dynamic Contrast-Enhanced Magnetic Resonance Imaging Study with Healthy Controls. Brain Behav. Immun..

[39] Murayama R., Cai Y., Nakamura H., Hashimoto K. (2025). Demyelination in Psychiatric and Neurological Disorders: Mechanisms, Clinical Impact, and Novel Therapeutic Strategies. Neurosci. Biobehav. Rev..

[40] Cheng Y., Wang T., Zhang T., Yi S., Zhao S., Li N., Yang Y., Zhang F., Xu L., Shan B. (2022). Increased Blood-Brain Barrier Permeability of the Thalamus Correlated with Symptom Severity and Brain Volume Alterations in Patients with Schizophrenia. Biol. Psychiatry Cogn. Neurosci. Neuroimaging.

[41] Che J., Sun Y., Deng Y., Zhang J. (2024). Blood-Brain Barrier Disruption: A Culprit of Cognitive Decline?. Fluids Barriers CNS.

[42] Wakonigg Alonso C., McElhatton F., O’Mahony B., Campbell M., Pollak T.A., Stokes P.R.A. (2024). The Blood-Brain Barrier in Bipolar Disorders: A Systematic Review. J. Affect. Disord..

[43] Zhang F., Zhang J., Wang X., Han M., Fei Y., Wang J. (2025). Blood–Brain Barrier Disruption in Schizophrenia: Insights, Mechanisms, and Future Directions. Int. J. Mol. Sci..

[44] Baethge C., Goldbeck-Wood S., Mertens S. (2019). SANRA-a Scale for the Quality Assessment of Narrative Review Articles. Res. Integr. Peer Rev..

[45] Kim S., Jung U.J., Kim S.R. (2024). Role of Oxidative Stress in Blood–Brain Barrier Disruption and Neurodegenerative Diseases. Antioxidants.

[46] Rajeev V., Fann D.Y., Dinh Q.N., Kim H.A., De Silva T.M., Lai M.K.P., Chen C.L.H., Drummond G.R., Sobey C.G., Arumugam T.V. (2022). Pathophysiology of Blood Brain Barrier Dysfunction during Chronic Cerebral Hypoperfusion in Vascular Cognitive Impairment. Theranostics.

[47] Chen T., Dai Y., Hu C., Lin Z., Wang S., Yang J., Zeng L., Li S., Li W. (2024). Cellular and Molecular Mechanisms of the Blood–Brain Barrier Dysfunction in Neurodegenerative Diseases. Fluids Barriers CNS.

[48] Wu S., Yin Y., Du L. (2021). Blood–Brain Barrier Dysfunction in the Pathogenesis of Major Depressive Disorder. Cell. Mol. Neurobiol..

[49] Li X., Ding W., Zhang X., Tang X., Zheng Y., Ma Y., Bao X., Kang X., Zhu S. (2025). Caveolin-1–Mediated Blood-Brain Barrier Disruption via MMP2/9 Contributes to Postoperative Cognitive Dysfunction. Neurochem. Res..

[50] Greene C., Hanley N., Campbell M. (2020). Blood-Brain Barrier Associated Tight Junction Disruption Is a Hallmark Feature of Major Psychiatric Disorders. Transl. Psychiatry.

[51] Candelario-Jalil E., Dijkhuizen R.M., Magnus T. (2022). Neuroinflammation, Stroke, Blood-Brain Barrier Dysfunction, and Imaging Modalities. Stroke.

[52] Wang Y., Liu W., Zhang J., Geng P., Jin X. (2024). The Role of Crosstalk between Cerebral Immune Cells and Peripheral Immune Cells in the Damage and Protection of Blood–Brain Barrier after Intracerebral Hemorrhage. Brain Hemorrhages.

[53] Yang J., Ran M., Li H., Lin Y., Ma K., Yang Y., Fu X., Yang S. (2022). New Insight into Neurological Degeneration: Inflammatory Cytokines and Blood–Brain Barrier. Front. Mol. Neurosci..

[54] Huang X., Hussain B., Chang J. (2020). Peripheral Inflammation and Blood–Brain Barrier Disruption: Effects and Mechanisms. CNS Neurosci. Ther..

[55] Acioglu C., Elkabes S. (2025). Innate Immune Sensors and Regulators at the Blood Brain Barrier: Focus on Toll-like Receptors and Inflammasomes as Mediators of Neuro-Immune Crosstalk and Inflammation. J. Neuroinflamm..

[56] Karwa P.N., Parekar V., Buttepatil S., Sakle N.S. (2026). Neuroinflammation as a Pharmacological Target in Psychiatric Disorders. Curr. Pharm. Des..

[57] Gao C., Jiang J., Tan Y., Chen S. (2023). Microglia in Neurodegenerative Diseases: Mechanism and Potential Therapeutic Targets. Signal Transduct. Target. Ther..

[58] Jung O., Thomas A., Burks S.R., Dustin M.L., Frank J.A., Ferrer M., Stride E. (2022). Neuroinflammation Associated with Ultrasound-Mediated Permeabilization of the Blood–Brain Barrier. Trends Neurosci..

[59] Kim S., Jung U.J., Kim S.R. (2025). The Crucial Role of the Blood–Brain Barrier in Neurodegenerative Diseases: Mechanisms of Disruption and Therapeutic Implications. J. Clin. Med..

[60] Takata F., Nakagawa S., Matsumoto J., Dohgu S. (2021). Blood-Brain Barrier Dysfunction Amplifies the Development of Neuroinflammation: Understanding of Cellular Events in Brain Microvascular Endothelial Cells for Prevention and Treatment of BBB Dysfunction. Front. Cell. Neurosci..

[61] Brandl S., Reindl M. (2023). Blood–Brain Barrier Breakdown in Neuroinflammation: Current In Vitro Models. Int. J. Mol. Sci..

[62] Vrillon A., Ashton N.J., Bouaziz-Amar E., Mouton-Liger F., Cognat E., Dumurgier J., Lilamand M., Karikari T.K., Prevot V., Zetterberg H. (2025). Dissection of Blood–Brain Barrier Dysfunction through CSF PDGFRβ and Amyloid, Tau, Neuroinflammation, and Synaptic CSF Biomarkers in Neurodegenerative Disorders. eBioMedicine.

[63] Millett C.E., Burdick K.E., Kubicki M.R. (2022). The Effects of Peripheral Inflammation on the Brain—A Neuroimaging Perspective. Harv. Rev. Psychiatry.

[64] Jeppesen R., Orlovska-Waast S., Sørensen N.V., Christensen R.H.B., Benros M.E. (2022). Cerebrospinal Fluid and Blood Biomarkers of Neuroinflammation and Blood-Brain Barrier in Psychotic Disorders and Individually Matched Healthy Controls. Schizophr. Bull..

[65] Medina-Rodriguez E.M., Beurel E. (2022). Blood Brain Barrier and Inflammation in Depression. Neurobiol. Dis..

[66] Ritson M., Wheeler-Jones C.P.D., Stolp H.B. (2024). Endothelial Dysfunction in Neurodegenerative Disease: Is Endothelial Inflammation an Overlooked Druggable Target?. J. Neuroimmunol..

[67] Goldsmith D.R., Rapaport M.H., Miller B.J. (2016). A Meta-Analysis of Blood Cytokine Network Alterations in Psychiatric Patients: Comparisons between Schizophrenia, Bipolar Disorder and Depression. Mol. Psychiatry.

[68] Osimo E.F., Baxter L.J., Lewis G., Jones P.B., Khandaker G.M. (2019). Prevalence of Low-Grade Inflammation in Depression: A Systematic Review and Meta-Analysis of CRP Levels. Psychol. Med..

[69] Wang A.K., Miller B.J. (2018). Meta-Analysis of Cerebrospinal Fluid Cytokine and Tryptophan Catabolite Alterations in Psychiatric Patients: Comparisons Between Schizophrenia, Bipolar Disorder, and Depression. Schizophr. Bull..

[70] Orlovska-Waast S., Köhler-Forsberg O., Brix S.W., Nordentoft M., Kondziella D., Krogh J., Benros M.E. (2019). Cerebrospinal Fluid Markers of Inflammation and Infections in Schizophrenia and Affective Disorders: A Systematic Review and Meta-Analysis. Mol. Psychiatry.

[71] Zhu Y., Webster M.J., Mendez Victoriano G., Middleton F.A., Massa P.T., Weickert C.S. (2025). Molecular Evidence for Altered Angiogenesis in Neuroinflammation-Associated Schizophrenia and Bipolar Disorder Implicate an Abnormal Midbrain Blood-Brain Barrier. Schizophr. Bull..

[72] Perry B.I., Upthegrove R., Kappelmann N., Jones P.B., Burgess S., Khandaker G.M. (2021). Associations of Immunological Proteins/Traits with Schizophrenia, Major Depression and Bipolar Disorder: A Bi-Directional Two-Sample Mendelian Randomization Study. Brain Behav. Immun..

[73] Menard C., Pfau M.L., Hodes G.E., Kana V., Wang V.X., Bouchard S., Takahashi A., Flanigan M.E., Aleyasin H., Leclair K.B. (2017). Social Stress Induces Neurovascular Pathology Promoting Depression. Nat. Neurosci..

[74] Dion-Albert L., Cadoret A., Doney E., Kaufmann F.N., Dudek K.A., Daigle B., Parise L.F., Cathomas F., Samba N., Hudson N. (2022). Vascular and Blood-Brain Barrier-Related Changes Underlie Stress Responses and Resilience in Female Mice and Depression in Human Tissue. Nat. Commun..

[75] Barrios L.C., da Silva Pinheiro A.P., Gibaldi D., Silva A.A., Rodrigues e Silva P.M., Roffê E., da Costa Santiago H., Gazzinelli R.T., Mineo J.R., Silva N.M. (2021). Behavioral Alterations in Long-Term Toxoplasma Gondii Infection of C57BL/6 Mice Are Associated with Neuroinflammation and Disruption of the Blood Brain Barrier. PLoS ONE.

[76] Zhang W., Xiao D., Mao Q., Xia H. (2023). Role of Neuroinflammation in Neurodegeneration Development. Signal Transduct. Target. Ther..

[77] Profaci C.P., Munji R.N., Pulido R.S., Daneman R. (2020). The Blood–Brain Barrier in Health and Disease: Important Unanswered Questions. J. Exp. Med..

[78] Beltran-Velasco A.I., Clemente-Suárez V.J. (2025). Impact of Peripheral Inflammation on Blood-Brain Barrier Dysfunction and Its Role in Neurodegenerative Diseases. Int. J. Mol. Sci..

[79] Alhayaza R., Haque E., Karbasiafshar C., Sellke F.W., Abid M.R. (2020). The Relationship Between Reactive Oxygen Species and Endothelial Cell Metabolism. Front. Chem..

[80] Feng J., Zheng Y., Guo M., Ares I., Martínez M., Lopez-Torres B., Martínez-Larrañaga M.R., Wang X., Anadón A., Martínez M.A. (2023). Oxidative Stress, the Blood–Brain Barrier and Neurodegenerative Diseases: The Critical Beneficial Role of Dietary Antioxidants. Acta Pharm. Sin. B.

[81] Wang Y., Wu J., Wang J., He L., Lai H., Zhang T., Wang X., Li W. (2023). Mitochondrial Oxidative Stress in Brain Microvascular Endothelial Cells: Triggering Blood-Brain Barrier Disruption. Mitochondrion.

[82] Liu S., Liu J., Wang Y., Deng F., Deng Z. (2025). Oxidative Stress: Signaling Pathways, Biological Functions, and Disease. MedComm.

[83] Jiménez-Fernández S., Gurpegui M., Garrote-Rojas D., Gutiérrez-Rojas L., Carretero M.D., Correll C.U. (2021). Oxidative Stress Parameters and Antioxidants in Patients with Bipolar Disorder: Results from a Meta-Analysis Comparing Patients, Including Stratification by Polarity and Euthymic Status, with Healthy Controls. Bipolar Disord..

[84] Flatow J., Buckley P., Miller B.J. (2013). Meta-Analysis of Oxidative Stress in Schizophrenia. Biol. Psychiatry.

[85] Black C.N., Bot M., Scheffer P.G., Cuijpers P., Penninx B.W.J.H. (2015). Is Depression Associated with Increased Oxidative Stress? A Systematic Review and Meta-Analysis. Psychoneuroendocrinology.

[86] Andreazza A.C., Shoo L., Wang J.F., Trevor Young L. (2010). Mitochondrial Complex I Activity and Oxidative Damage to Mitochondrial Proteins in the Prefrontal Cortex of Patients with Bipolar Disorder. Arch. Gen. Psychiatry.

[87] Haileselassie B., Joshi A.U., Minhas P.S., Mukherjee R., Andreasson K.I., Mochly-Rosen D. (2020). Mitochondrial Dysfunction Mediated through Dynamin-Related Protein 1 (Drp1) Propagates Impairment in Blood Brain Barrier in Septic Encephalopathy. J. Neuroinflamm..

[88] Cheng X., Potenza D.M., Brenna A., Ajalbert G., Yang Z., Ming X.F. (2024). Aging Increases Hypoxia-Induced Endothelial Permeability and Blood-Brain Barrier Dysfunction by Upregulating Arginase-II. Aging Dis..

[89] Chen J.X.Y., Vipin A., Sandhu G.K., Leow Y.J., Zailan F.Z., Tanoto P., Lee E.S., Lee K.L., Cheung C., Kandiah N. (2025). Blood-Brain Barrier Integrity Disruption Is Associated with Both Chronic Vascular Risk Factors and White Matter Hyperintensities. J. Prev. Alzheimer’s Dis..

[90] Cyr A.R., Huckaby L.V., Shiva S.S., Zuckerbraun B.S. (2020). Nitric Oxide and Endothelial Dysfunction. Crit. Care Clin..

[91] Raza A., Saleem S., Imran S., Rahman S., Haroon M., Razzaq A., Hussain A., Iqbal J., Sathian B. (2025). From Metabolic Dysregulation to Neurodegenerative Pathology: The Role of Hyperglycemia, Oxidative Stress, and Blood-Brain Barrier Breakdown in T2D-Driven Alzheimer’s Disease. Metab. Brain Dis..

[92] Puvogel S., Palma V., Sommer I.E.C. (2022). Brain Vasculature Disturbance in Schizophrenia. Curr. Opin. Psychiatry.

[93] González P., Lozano P., Ros G., Solano F. (2023). Hyperglycemia and Oxidative Stress: An Integral, Updated and Critical Overview of Their Metabolic Interconnections. Int. J. Mol. Sci..

[94] Kamintsky L., Cairns K.A., Veksler R., Bowen C., Beyea S.D., Friedman A., Calkin C. (2020). Blood-Brain Barrier Imaging as a Potential Biomarker for Bipolar Disorder Progression. Neuroimage Clin..

[95] Toma S., MacIntosh B.J., Swardfager W., Goldstein B.I. (2018). Cerebral Blood Flow in Bipolar Disorder: A Systematic Review. J. Affect. Disord..

[96] Zhao N., Chung T.D., Guo Z., Jamieson J.J., Liang L., Linville R.M., Pessell A.F., Wang L., Searson P.C. (2023). The Influence of Physiological and Pathological Perturbations on Blood-Brain Barrier Function. Front. Neurosci..

[97] Sher L.D., Geddie H., Olivier L., Cairns M., Truter N., Beselaar L., Essop M.F. (2020). Chronic Stress and Endothelial Dysfunction: Mechanisms, Experimental Challenges, and the Way Ahead. Am. J. Physiol.-Heart Circ. Physiol..

[98] Förster C., Burek M., Romero I.A., Weksler B., Couraud P.O., Drenckhahn D. (2008). Differential Effects of Hydrocortisone and TNFalpha on Tight Junction Proteins in an in Vitro Model of the Human Blood-Brain Barrier. J. Physiol..

[99] Förster C., Silwedel C., Golenhofen N., Burek M., Kietz S., Mankertz J., Drenckhahn D. (2005). Occludin as Direct Target for Glucocorticoid-Induced Improvement of Blood-Brain Barrier Properties in a Murine in Vitro System. J. Physiol..

[100] Taylor M.A., Kokiko-Cochran O.N. (2024). Context Is Key: Glucocorticoid Receptor and Corticosteroid Therapeutics in Outcomes after Traumatic Brain Injury. Front. Cell. Neurosci..

[101] Gadwala S., Ghosh C. (2025). The Role of the Glucocorticoid Receptor and Its Phosphorylation in Neurological Disorders. Int. J. Mol. Sci..

[102] Fabbri C. (2022). Genetics in Psychiatry: Methods, Clinical Applications and Future Perspectives. Psychiatry Clin. Neurosci. Rep..

[103] Sălcudean A., Bodo C.R., Popovici R.A., Cozma M.M., Păcurar M., Crăciun R.E., Crisan A.I., Enatescu V.R., Marinescu I., Cimpian D.M. (2025). Neuroinflammation—A Crucial Factor in the Pathophysiology of Depression—A Comprehensive Review. Biomolecules.

[104] Nestler E.J., Peña C.J., Kundakovic M., Mitchell A., Akbarian S. (2015). Epigenetic Basis of Mental Illness. Neuroscientist.

[105] Hakami M.A. (2025). Molecular Signatures and Emerging Therapeutic Targets in Schizophrenia: A Biomarker-Centric Perspective in Psychiatric Disorders. Schizophr. Res..

[106] Malhi G.S., Mann J.J. (2018). Depression. Lancet.

[107] Anderson E., Crawford C.M., Fava M., Ingelfinger J., Nikayin S., Sanacora G., Scott-Vernaglia S., Teel J. (2024). Depression—Understanding, Identifying, and Diagnosing. N. Engl. J. Med..

[108] Simon G.E., Moise N., Mohr D.C. (2024). Management of Depression in Adults: A Review. JAMA.

[109] Wu L., Wu F., Li M., Liu Y., Wang S., Guo S., Liu S., Jiang X., Zhou Y., Kong L. (2025). The Elevated Plasma Levels of Claudin-5 Are Associated with Peripheral Inflammatory Activity in Patients with Major Depressive Disorder. J. Affect. Disord..

[110] Dudek K.A., Dion-Albert L., Lebel M., LeClair K., Labrecque S., Tuck E., Perez C.F., Golden S.A., Tamminga C., Turecki G. (2020). Molecular Adaptations of the Blood–Brain Barrier Promote Stress Resilience vs. Depression. Proc. Natl. Acad. Sci. USA.

[111] Matsuno H., Tsuchimine S., O’Hashi K., Sakai K., Hattori K., Hidese S., Nakajima S., Chiba S., Yoshimura A., Fukuzato N. (2022). Association between Vascular Endothelial Growth Factor-Mediated Blood-Brain Barrier Dysfunction and Stress-Induced Depression. Mol. Psychiatry.

[112] Dudek K.A., Paton S.E.J., Binder L.B., Collignon A., Dion-Albert L., Cadoret A., Lebel M., Lavoie O., Bouchard J., Kaufmann F.N. (2025). Astrocytic Cannabinoid Receptor 1 Promotes Resilience by Dampening Stress-Induced Blood-Brain Barrier Alterations. Nat. Neurosci..

[113] Nagy T., Baska D., Petschner P., Gonda X., Gál Z., Juhasz G., Bagdy G. (2024). Investigating the Role of TNF-Alpha through Blood-Brain Barrier Integrity in Stress-Induced Depression. Neuropsychopharmacol. Hung..

[114] Dang R., Wang M., Li X., Wang H., Liu L., Wu Q., Zhao J., Ji P., Zhong L., Licinio J. (2022). Edaravone Ameliorates Depressive and Anxiety-like Behaviors via Sirt1/Nrf2/HO-1/Gpx4 Pathway. J. Neuroinflamm..

[115] Tóth A.E., Walter F.R., Bocsik A., Sántha P., Veszelka S., Nagy L., Puskás L.G., Couraud P.O., Takata F., Dohgu S. (2014). Edaravone Protects against Methylglyoxal-Induced Barrier Damage in Human Brain Endothelial Cells. PLoS ONE.

[116] Yu X., Bai Y., Han B., Ju M., Tang T., Shen L., Li M., Yang L., Zhang Z., Hu G. (2022). Extracellular Vesicle-Mediated Delivery of CircDYM Alleviates CUS-Induced Depressive-like Behaviours. J. Extracell. Vesicles.

[117] Carvalho A.F., Firth J., Vieta E. (2020). Bipolar Disorder. N. Engl. J. Med..

[118] Singh B., Swartz H.A., Cuellar-Barboza A.B., Schaffer A., Kato T., Dols A., Sperry S.H., Vassilev A.B., Burdick K.E., Frye M.A. (2025). Bipolar Disorder. Lancet.

[119] Zhao N.O., Topolski N., Tusconi M., Salarda E.M., Busby C.W., Lima C.N.N.C., Pillai A., Quevedo J., Barichello T., Fries G.R. (2022). Blood-Brain Barrier Dysfunction in Bipolar Disorder: Molecular Mechanisms and Clinical Implications. Brain Behav. Immun. Health.

[120] Barger S.W., Van Eldik L.J. (1992). S100 Beta Stimulates Calcium Fluxes in Glial and Neuronal Cells. J. Biol. Chem..

[121] Janigro D., Bailey D.M., Lehmann S., Badaut J., O’Flynn R., Hirtz C., Marchi N. (2021). Peripheral Blood and Salivary Biomarkers of Blood-Brain Barrier Permeability and Neuronal Damage: Clinical and Applied Concepts. Front. Neurol..

[122] Gnyliukh N., Wei J., Neuhaus W., Boukherroub R., Szunerits S. (2025). The Role of S100B Protein as a Diagnostic Biomarker for Brain Injury. Sens. Biosensing Res..

[123] Millett C.E., Monir F., Sanelli P. (2025). The Role of Blood-Brain Barrier Dysfunction in Cognitive Impairments in Bipolar Disorder-a Narrative Review. Front. Hum. Neurosci..

[124] Hsu J.W., Chen L.C., Bai Y.M., Tsai S.J., Chen M.H. (2025). Distinct Effects of Psychiatric Disorder Diagnoses and Severe Emotional Dysregulation on Matrix Metalloproteinase-9, Proinflammatory Cytokines, and Inhibitory Control Function in Adolescents with Attention-Deficit Hyperactivity Disorder or First-Episode Major Affective Disorders. Int. J. Neuropsychopharmacol..

[125] Dickerson F., Vaidya D., Liu Y., Yolken R. (2023). Levels of Matrix Metalloproteinase 9 Are Elevated in Persons with Schizophrenia or Bipolar Disorder: The Role of Modifiable Factors. Biol. Psychiatry Glob. Open Sci..

[126] Calkin C., McClelland C., Cairns K., Kamintsky L., Friedman A. (2021). Insulin Resistance and Blood-Brain Barrier Dysfunction Underlie Neuroprogression in Bipolar Disorder. Front. Psychiatry.

[127] Rinaldi C., Donato L., Alibrandi S., Scimone C., D’angelo R., Sidoti A. (2021). Oxidative Stress and the Neurovascular Unit. Life.

[128] Barichello T., Giridharan V.V., Bhatti G., Sayana P., Doifode T., Macedo D., Quevedo J. (2021). Inflammation as a Mechanism of Bipolar Disorder Neuroprogression. Curr. Top. Behav. Neurosci..

[129] Wollenhaupt-Aguiar B., Kapczinski F., Pfaffenseller B. (2021). Biological Pathways Associated with Neuroprogression in Bipolar Disorder. Brain Sci..

[130] Calkin C., Kamintsky L., Friedman A. (2022). Reversal of Insulin Resistance Is Associated with Repair of Blood-Brain Barrier Dysfunction and Remission in a Patient with Treatment-Resistant Bipolar Depression. Bipolar Disord..

[131] Marder S.R., Cannon T.D. (2019). Schizophrenia. N. Engl. J. Med..

[132] Jauhar S., Johnstone M., McKenna P.J. (2022). Schizophrenia. Lancet.

[133] McCutcheon R.A., Pillinger T., Varvari I., Halstead S., Ayinde O.O., Crossley N.A., Correll C.U., Hahn M., Howes O.D., Kane J.M. (2025). INTEGRATE: International Guidelines for the Algorithmic Treatment of Schizophrenia. Lancet Psychiatry.

[134] Campana M., Yakimov V., Moussiopoulou J., Maurus I., Löhrs L., Raabe F., Jäger I., Mortazavi M., Benros M.E., Jeppesen R. (2024). Association of Symptom Severity and Cerebrospinal Fluid Alterations in Recent Onset Psychosis in Schizophrenia-Spectrum Disorders—An Individual Patient Data Meta-Analysis. Brain Behav. Immun..

[135] Konen F.F., Gehring P.S., Maier H.B., Schröder S., Türker S.N., Frieling H., Bleich S., Huss A., Tumani H., Lüdecke D. (2025). Pilot Study of Cerebrospinal Fluid Biomarkers Reveals Inflammatory Changes in Patients with Paranoid Schizophrenia. Sci. Rep..

[136] Yang H.M. (2025). Overcoming the Blood–Brain Barrier: Advanced Strategies in Targeted Drug Delivery for Neurodegenerative Diseases. Pharmaceutics.

[137] Al Rihani S.B., Batarseh Y.S., Kaddoumi A. (2023). The Blood–Brain Barrier in Health and Disease. Int. J. Mol. Sci..

[138] Zhang X., Valeri J., Eladawi M.A., Gisabella B., Garrett M.R., Vallender E.J., McCullumsmith R., Pantazopoulos H., O’Donovan S.M. (2026). Transcriptomic Analysis of the Amygdala in Subjects with Schizophrenia, Bipolar Disorder and Major Depressive Disorder Reveals Differentially Altered Metabolic Pathways. Schizophr. Bull..

[139] Stankovic I., Notaras M., Wolujewicz P., Lu T., Lis R., Ross M.E., Colak D. (2024). Schizophrenia Endothelial Cells Exhibit Higher Permeability and Altered Angiogenesis Patterns in Patient-Derived Organoids. Transl. Psychiatry.

[140] Zhang Y. (2025). Research Hotspots and Emerging Trends of Schizophrenia and Immune Response: A Bibliometric Analysis. Brain Behav..

[141] Sweeney M.D., Ayyadurai S., Zlokovic B.V. (2016). Pericytes of the Neurovascular Unit: Key Functions and Signaling Pathways. Nat. Neurosci..

[142] Li J., Zheng M., Shimoni O., Banks W.A., Bush A.I., Gamble J.R., Shi B. (2021). Development of Novel Therapeutics Targeting the Blood–Brain Barrier: From Barrier to Carrier. Adv. Sci..

[143] Fu X., Li J., Yang S., Jing J., Zheng Q., Zhang T., Xu Z. (2025). Blood-Brain Barrier Repair: Potential and Challenges of Stem Cells and Exosomes in Stroke Treatment. Front. Cell. Neurosci..

[144] Dash U.C., Bhol N.K., Swain S.K., Samal R.R., Nayak P.K., Raina V., Panda S.K., Kerry R.G., Duttaroy A.K., Jena A.B. (2025). Oxidative Stress and Inflammation in the Pathogenesis of Neurological Disorders: Mechanisms and Implications. Acta Pharm. Sin. B.

[145] Zhao L., Hou C., Yan N. (2022). Neuroinflammation in Retinitis Pigmentosa: Therapies Targeting the Innate Immune System. Front. Immunol..

[146] Mohapatra L., Mishra D., Tripathi A.S., Parida S.K., Palei N.N. (2025). Illustrating the Pathogenesis and Therapeutic Approaches of Epilepsy by Targeting Angiogenesis, Inflammation, and Oxidative Stress. Neuroglia.

[147] Szkudlarek H.J., Singh Mann R., Wieczerzak K., Sarikahya M.H., Uzuneser T.C., De Felice M., Rodríguez-Ruiz M., Galindo J.P., Pusparajah M., Whitehead S.N. (2025). The Antioxidant N-Acetylcysteine Prevents Cortical Neuropathological Phenotypes Caused by Adolescent Δ-9-Tetrahydrocannabinol Exposure in Male Rats. Transl. Psychiatry.

[148] Cherneva D.I., Kehayova G., Dimitrova S., Dragomanova S. (2025). The Central Nervous System Modulatory Activities of N-Acetylcysteine: A Synthesis of Two Decades of Evidence. Curr. Issues Mol. Biol..

[149] Kawoos U., Abutarboush R., Zarriello S., Qadri A., Ahlers S.T., McCarron R.M., Chavko M. (2019). N-Acetylcysteine Amide Ameliorates Blast-Induced Changes in Blood-Brain Barrier Integrity in Rats. Front. Neurol..

[150] Lin J., Yang F., Hu A., Tang X., Lin L., Wu P., Kong H., Liu C., Cui K., Yang B. (2025). N-Acetylcysteine Amide Alleviates Pathological Neovascularization through Mitochondrial Protection and Energy Metabolism Reprogramming of Vascular Endothelial Cells. Redox Biol..

[151] Behl T., Rana T., Alotaibi G.H., Shamsuzzaman M., Naqvi M., Sehgal A., Singh S., Sharma N., Almoshari Y., Abdellatif A.A.H. (2022). Polyphenols Inhibiting MAPK Signalling Pathway Mediated Oxidative Stress and Inflammation in Depression. Biomed. Pharmacother..

[152] Marasinghe C.K., Suryaningtyas I.T., Jung W.K., Je J.Y. (2025). Protective Effect of N-Acetylcysteine (NAC) on OxLDL-Induced Endothelial Dysfunction. J. Microbiol. Biotechnol..

[153] Trofin D.M., Sardaru D.P., Trofin D., Onu I., Tutu A., Onu A., Onită C., Galaction A.I., Matei D.V. (2025). Oxidative Stress in Brain Function. Antioxidants.

[154] Kim Y., Cho A.Y., Kim H.C., Ryu D., Jo S.A., Jung Y.S. (2022). Effects of Natural Polyphenols on Oxidative Stress-Mediated Blood-Brain Barrier Dysfunction. Antioxidants.

[155] Soda T., Brunetti V., De Sarro G., Biella G., Moccia F., Berra-Romani R., Scarpellino G. (2025). Transient Receptor Potential Ankyrin 1 (TRPA1) Mediates Hydrogen Sulfide-Induced Ca^2+^ Entry and Nitric Oxide Production in Human Cerebrovascular Endothelium. Curr. Neuropharmacol..

[156] Nasyrova R.F., Ivashchenko D.V., Ivanov M.V., Neznanov N.G. (2015). Role of Nitric Oxide and Related Molecules in Schizophrenia Pathogenesis: Biochemical, Genetic and Clinical Aspects. Front. Physiol..

[157] Zoupa E., Pitsikas N., Maccallini C., Amoroso R. (2021). The Nitric Oxide (NO) Donor Sodium Nitroprusside (SNP) and Its Potential for the Schizophrenia Therapy: Lights and Shadows. Molecules.

[158] Xiong J.W., Wei B., Li Y.K., Zhan J.Q., Jiang S.Z., Chen H.B., Yan K., Yu B., Yang Y.J. (2018). Decreased Plasma Levels of Gasotransmitter Hydrogen Sulfide in Patients with Schizophrenia: Correlation with Psychopathology and Cognition. Psychopharmacology.

[159] Cao S., Zhu P., Yu X., Chen J., Li J., Yan F., Wang L., Yu J., Chen G. (2016). Hydrogen Sulfide Attenuates Brain Edema in Early Brain Injury after Subarachnoid Hemorrhage in Rats: Possible Involvement of MMP-9 Induced Blood-Brain Barrier Disruption and AQP4 Expression. Neurosci. Lett..

[160] Li H., Zhu L., Feng J., Hu X., Li C., Zhang B. (2018). Hydrogen Sulfide Decreases Blood-Brain Barrier Damage via Regulating Protein Kinase C and Tight Junction After Cardiac Arrest in Rats. Cell. Physiol. Biochem..

[161] Lee Y., Mansur R.B., Brietzke E., Carmona N.E., Subramaniapillai M., Pan Z., Shekotikhina M., Rosenblat J.D., Suppes T., Cosgrove V.E. (2020). Efficacy of Adjunctive Infliximab vs. Placebo in the Treatment of Anhedonia in Bipolar I/II Depression. Brain Behav. Immun..

[162] Panizzutti B., Skvarc D., Lin S., Croce S., Meehan A., Bortolasci C.C., Marx W., Walker A.J., Hasebe K., Kavanagh B.E. (2023). Minocycline as Treatment for Psychiatric and Neurological Conditions: A Systematic Review and Meta-Analysis. Int. J. Mol. Sci..

[163] Gong H., Su W.J., Deng S.L., Luo J., Du Z.L., Luo Y., Lv K.Y., Zhu D.M., Fan X.T. (2025). Anti-Inflammatory Interventions for the Treatment and Prevention of Depression among Older Adults: A Systematic Review and Meta-Analysis. Transl. Psychiatry.

[164] Perrone M.G., Centonze A., Miciaccia M., Ferorelli S., Scilimati A. (2020). Cyclooxygenase Inhibition Safety and Efficacy in Inflammation-Based Psychiatric Disorders. Molecules.

[165] Müller N. (2019). COX-2 Inhibitors, Aspirin, and Other Potential Anti-Inflammatory Treatments for Psychiatric Disorders. Front. Psychiatry.

[166] Köhler-Forsberg O., Lydholm C.N., Hjorthøj C., Nordentoft M., Mors O., Benros M.E. (2019). Efficacy of Anti-Inflammatory Treatment on Major Depressive Disorder or Depressive Symptoms: Meta-Analysis of Clinical Trials. Acta Psychiatr. Scand..

[167] Nery F.G., Monkul E.S., Hatch J.P., Fonseca M., Zunta-Soares G.B., Frey B.N., Bowden C.L., Soares J.C. (2008). Celecoxib as an Adjunct in the Treatment of Depressive or Mixed Episodes of Bipolar Disorder: A Double-Blind, Randomized, Placebo-Controlled Study. Hum. Psychopharmacol..

[168] Nassar A., Azab A.N. (2014). Effects of Lithium on Inflammation. ACS Chem. Neurosci..

[169] Uzzan S., Azab A.N. (2021). Anti-TNF-α Compounds as a Treatment for Depression. Molecules.

[170] Müller N., Riedel M., Scheppach C., Brandstätter B., Sokullu S., Krampe K., Ulmschneider M., Engel R.R., Möller H.J., Schwarz M.J. (2002). Beneficial Antipsychotic Effects of Celecoxib Add-on Therapy Compared to Risperidone Alone in Schizophrenia. Am. J. Psychiatry.

[171] Husain M.I., Chaudhry I.B., Khoso A.B., Husain M.O., Hodsoll J., Ansari M.A., Naqvi H.A., Minhas F.A., Carvalho A.F., Meyer J.H. (2020). Minocycline and Celecoxib as Adjunctive Treatments for Bipolar Depression: A Multicentre, Factorial Design Randomised Controlled Trial. Lancet Psychiatry.

[172] Faridhosseini F., Talaei A., Shahini N., Salimi Z., Eslamzadeh M., Ahrari S., Pourgholami M., Khadem-Rezaiyan M. (2023). Does Celecoxib with Sodium Valproate Have an Augmentation Effect on Acute Mania in Bipolar Disorder? A Double-Blind Controlled Clinical Trial in Iran. Int. Clin. Psychopharmacol..

[173] Sampson E., Mills N.T., Hori H., Schwarte K., Hohoff C., Schubert K.O., Clark S.R., Fourrier C., Baune B.T. (2023). Exploratory Analysis of the Effects of Celecoxib on Cognitive Function in Vortioxetine-Treated Patients with Major Depressive Disorder in the PREDDICT Study: A Randomized, Double-Blind, Placebo-Controlled Clinical Trial. J. Clin. Psychiatry.

[174] Baune B.T., Sampson E., Louise J., Hori H., Schubert K.O., Clark S.R., Mills N.T., Fourrier C. (2021). No Evidence for Clinical Efficacy of Adjunctive Celecoxib with Vortioxetine in the Treatment of Depression: A 6-Week Double-Blind Placebo Controlled Randomized Trial. Eur. Neuropsychopharmacol..

[175] McIntyre R.S., Subramaniapillai M., Lee Y., Pan Z., Carmona N.E., Shekotikhina M., Rosenblat J.D., Brietzke E., Soczynska J.K., Cosgrove V.E. (2019). Efficacy of Adjunctive Infliximab vs Placebo in the Treatment of Adults with Bipolar I/II Depression: A Randomized Clinical Trial. JAMA Psychiatry.

[176] Raison C.L., Rutherford R.E., Woolwine B.J., Shuo C., Schettler P., Drake D.F., Haroon E., Miller A.H. (2013). A Randomized Controlled Trial of the Tumor Necrosis Factor Antagonist Infliximab for Treatment-Resistant Depression: The Role of Baseline Inflammatory Biomarkers. JAMA Psychiatry.

[177] Gryka-Marton M., Grabowska A.D., Szukiewicz D. (2025). Breaking the Barrier: The Role of Proinflammatory Cytokines in BBB Dysfunction. Int. J. Mol. Sci..

[178] Mallick R., Basak S., Chowdhury P., Bhowmik P., Das R.K., Banerjee A., Paul S., Pathak S., Duttaroy A.K. (2025). Targeting Cytokine-Mediated Inflammation in Brain Disorders: Developing New Treatment Strategies. Pharmaceuticals.

[179] Anaya-Prado R., Canseco-Villegas A.I., Anaya-Fernández R., Anaya-Fernandez M.M., Guerrero-Palomera M.A., Guerrero-Palomera C., Garcia-Ramirez I.F., Gonzalez-Martinez D., Azcona-Ramírez C.C., Garcia-Perez C. (2025). Role of Nitric Oxide in Cerebral Ischemia/Reperfusion Injury: A Biomolecular Overview. World J. Clin. Cases.

[180] Scarpellino G., Brunetti V., Berra-Romani R., De Sarro G., Guerra G., Soda T., Moccia F. (2024). The Unexpected Role of the Endothelial Nitric Oxide Synthase at the Neurovascular Unit: Beyond the Regulation of Cerebral Blood Flow. Int. J. Mol. Sci..

[181] Sarmah N., Nauli A.M., Ally A., Nauli S.M. (2022). Interactions among Endothelial Nitric Oxide Synthase, Cardiovascular System, and Nociception during Physiological and Pathophysiological States. Molecules.

[182] Hosseini N., Kourosh-Arami M., Nadjafi S., Ashtari B. (2022). Structure, Distribution, Regulation, and Function of Splice Variant Isoforms of Nitric Oxide Synthase Family in the Nervous System. Curr. Protein Pept. Sci..

[183] Crabtree M.J., Tatham A.L., Al-Wakeel Y., Warrick N., Hale A.B., Cai S., Channon K.M., Alp N.J. (2009). Quantitative Regulation of Intracellular Endothelial Nitric-Oxide Synthase (ENOS) Coupling by Both Tetrahydrobiopterin-ENOS Stoichiometry and Biopterin Redox Status. J. Biol. Chem..

[184] Alp N.J., Channon K.M. (2004). Regulation of Endothelial Nitric Oxide Synthase by Tetrahydrobiopterin in Vascular Disease. Arterioscler. Thromb. Vasc. Biol..

[185] Chen W.H., Chen C.H., Hsu M.C., Chang R.W., Wang C.H., Lee T.S. (2024). Advances in the Molecular Mechanisms of Statins in Regulating Endothelial Nitric Oxide Bioavailability: Interlocking Biology between ENOS Activity and L-Arginine Metabolism. Biomed. Pharmacother..

[186] Laufs U. (2003). Beyond Lipid-Lowering: Effects of Statins on Endothelial Nitric Oxide. Eur. J. Clin. Pharmacol..

[187] Benicky J., Sánchez-Lemus E., Pavel J., Saavedra J.M. (2009). Anti-Inflammatory Effects of Angiotensin Receptor Blockers in the Brain and the Periphery. Cell. Mol. Neurobiol..

[188] Bar-Klein G., Lublinsky S., Kamintsky L., Noyman I., Veksler R., Dalipaj H., Senatorov V.V., Swissa E., Rosenbach D., Elazary N. (2017). Imaging Blood-Brain Barrier Dysfunction as a Biomarker for Epileptogenesis. Brain.

[189] Najjar S., Pahlajani S., De Sanctis V., Stern J.N.H., Najjar A., Chong D. (2017). Neurovascular Unit Dysfunction and Blood–Brain Barrier Hyperpermeability Contribute to Schizophrenia Neurobiology: A Theoretical Integration of Clinical and Experimental Evidence. Front. Psychiatry.

[190] Zhang X., Wang Y., Xue S., Gong L., Yan J., Zheng Y., Yang X., Fan Y., Han K., Chen Y. (2024). Chronic Stress in Adulthood Results in Microvascular Dysfunction and Subsequent Depressive-like Behavior. Sci. Rep..

[191] García R.G., Zarruk J.G., Barrera C., Pinzón A., Trillos E., Arenas W.D., Luengas C., Tomaz C., López-Jaramillo P. (2011). Plasma Nitrate Levels and Flow-Mediated Vasodilation in Untreated Major Depression. Psychosom. Med..

[192] Ikenouchi-Sugita A., Yoshimura R., Hori H., Umene-Nakano W., Ueda N., Nakamura J. (2009). Effects of Antidepressants on Plasma Metabolites of Nitric Oxide in Major Depressive Disorder: Comparison between Milnacipran and Paroxetine. Prog. Neuropsychopharmacol. Biol. Psychiatry.

[193] Ramirez-Velez I., Belardi B. (2023). Storming the Gate: New Approaches for Targeting the Dynamic Tight Junction for Improved Drug Delivery. Adv. Drug Deliv. Rev..

[194] Cha H.J. (2025). Tight Junction Proteins at the Crossroads of Inflammation, Barrier Function, and Disease Modulation. Int. J. Mol. Sci..

[195] Huang X., Wei P., Fang C., Yu M., Yang S., Qiu L., Wang Y., Xu A., Hoo R.L.C., Chang J. (2024). Compromised Endothelial Wnt/β-Catenin Signaling Mediates the Blood-Brain Barrier Disruption and Leads to Neuroinflammation in Endotoxemia. J. Neuroinflamm..

[196] Liu J., Xiao Q., Xiao J., Niu C., Li Y., Zhang X., Zhou Z., Shu G., Yin G. (2022). Wnt/β-Catenin Signalling: Function, Biological Mechanisms, and Therapeutic Opportunities. Signal Transduct. Target. Ther..

[197] Wang L., Li J., Di L. (2022). jun Glycogen Synthesis and beyond, a Comprehensive Review of GSK3 as a Key Regulator of Metabolic Pathways and a Therapeutic Target for Treating Metabolic Diseases. Med. Res. Rev..

[198] Snitow M.E., Bhansali R.S., Klein P.S. (2021). Lithium and Therapeutic Targeting of GSK-3. Cells.

[199] Luo H., Chevillard L., Bellivier F., Mégarbane B., Etain B., Cisternino S., Declèves X. (2021). The Role of Brain Barriers in the Neurokinetics and Pharmacodynamics of Lithium. Pharmacol. Res..

[200] Song D., Ji Y.B., Huang X.W., Ma Y.Z., Fang C., Qiu L.H., Tan X.X., Chen Y.M., Wang S.N., Chang J. (2022). Lithium Attenuates Blood–Brain Barrier Damage and Brain Edema Following Intracerebral Hemorrhage via an Endothelial Wnt/Β-catenin Signaling-dependent Mechanism in Mice. CNS Neurosci. Ther..

[201] Palla M., Scarpato L., Di Trolio R., Ascierto P.A. (2022). Sonic Hedgehog Pathway for the Treatment of Inflammatory Diseases: Implications and Opportunities for Future Research. J. Immunother. Cancer.

[202] Maridaki Z., Syrros G., Gianna Delichatsiou S., Warsh J., Konstantinou G.N. (2025). Claudin-5 and Occludin Levels in Patients with Psychiatric Disorders—A Systematic Review. Brain Behav. Immun..

[203] Hue C.D., Cho F.S., Cao S., Dale Bass C.R., Meaney D.F., Morrison B. (2015). Dexamethasone Potentiates in Vitro Blood-Brain Barrier Recovery after Primary Blast Injury by Glucocorticoid Receptor-Mediated Upregulation of ZO-1 Tight Junction Protein. J. Cereb. Blood Flow Metab..

[204] Stewart E.A., Saker S., Amoaku W.M. (2016). Dexamethasone Reverses the Effects of High Glucose on Human Retinal Endothelial Cell Permeability and Proliferation in Vitro. Exp. Eye Res..

[205] Na W., Shin J.Y., Lee J.Y., Jeong S., Kim W.S., Yune T.Y., Ju B.G. (2017). Dexamethasone Suppresses JMJD3 Gene Activation via a Putative Negative Glucocorticoid Response Element and Maintains Integrity of Tight Junctions in Brain Microvascular Endothelial Cells. J. Cereb. Blood Flow Metab..

[206] Zhao J., Shan Y., Cen Y., Wang M., Zhuang T., Li Y., Liu Y., Nie Z., Xie J., Zhang J. (2026). Effect of Different Doses of Dexamethasone on the Penetration of Acyclovir across the Blood-Brain Barrier. Eur. J. Pharm. Sci..

[207] Mehdizadeh S., Mamaghani M., Hassanikia S., Pilehvar Y., Ertas Y.N. (2025). Exosome-Powered Neuropharmaceutics: Unlocking the Blood-Brain Barrier for next-Gen Therapies. J. Nanobiotechnol..

[208] Mittal K.R., Pharasi N., Sarna B., Singh M., Rachana, Haider S., Singh S.K., Dua K., Jha S.K., Dey A. (2022). Nanotechnology-Based Drug Delivery for the Treatment of CNS Disorders. Transl. Neurosci..

[209] Kulkarni M., Patel K., Patel A., Patel S., Desai J., Patel M., Shah U., Patel A., Solanki N. (2023). Nanomaterials as Drug Delivery Agents for Overcoming the Blood-Brain Barrier: A Comprehensive Review. ADMET DMPK.

[210] Charabati M., Rabanel J.M., Ramassamy C., Prat A. (2020). Overcoming the Brain Barriers: From Immune Cells to Nanoparticles. Trends Pharmacol. Sci..

[211] Deng K., Chen Z., Zhang X., Li Y., Cheng F., Wang Z., Wang C. (2025). Harnessing Engineered Exosomes for Transformative Therapy of Cardiovascular and Cerebrovascular Disorders: Opportunities and Challenges. Mater. Des..

[212] Zhang X., Artz N., Steindler D.A., Hingtgen S., Satterlee A.B. (2025). Exosomes: Traversing the Blood-Brain Barrier and Their Therapeutic Potential in Brain Cancer. Biochim. Biophys. Acta (BBA)—Rev. Cancer.

[213] Sun M., Qin F., Bu Q., Zhao Y., Yang X., Zhang D., Cen X. (2025). Exosome-Based Therapeutics: A Natural Solution to Overcoming the Blood–Brain Barrier in Neurodegenerative Diseases. MedComm.

[214] Sanadgol N., Abedi M., Hashemzaei M., Kamran Z., Khalseh R., Beyer C., Voelz C. (2025). Exosomes as Nanocarriers for Brain-Targeted Delivery of Therapeutic Nucleic Acids: Advances and Challenges. J. Nanobiotechnol..

[215] Zhao P., Wu T., Tian Y., You J., Cui X. (2024). Recent Advances of Focused Ultrasound Induced Blood-Brain Barrier Opening for Clinical Applications of Neurodegenerative Diseases. Adv. Drug Deliv. Rev..

[216] De Maio A., Huang Y., Lin F.H., Stefanovic B., Stanisz G.J., O’Reilly M.A. (2025). Evaluation of Focused Ultrasound Modulation of the Blood-Brain Barrier in Gray and White Matter. J. Control. Release.

[217] Jiang P., Li J. (2025). Recent Advances in Biomimetic Nanodelivery Systems for the Treatment of Depression. Mater. Today Bio.

[218] Niazi S.K. (2023). Non-Invasive Drug Delivery across the Blood–Brain Barrier: A Prospective Analysis. Pharmaceutics.

[219] Konig S., Jayarajan V., Wray S., Kamm R., Moeendarbary E. (2025). Mechanobiology of the Blood-Brain Barrier during Development, Disease and Ageing. Nat. Commun..

[220] Adzic M., Lukic I., Mitic M., Zerajic E.F., Glavonic E., Jovanovic M., Ivkovic S. (2025). Nutritional Modulation of Impaired Blood-Brain Barrier Integrity and Function in Major Depression. Int. J. Mol. Sci..

[221] Kocsis A.E., Kucsápszky N., Santa-Maria A.R., Hunyadi A., Deli M.A., Walter F.R. (2025). Much More than Nutrients: The Protective Effects of Nutraceuticals on the Blood–Brain Barrier in Diseases. Nutrients.

[222] Yue R., Wen H., He L., Liu Y., Liang Z., Luo L. (2025). Tea and Blood–Brain Barrier Homeostasis: Potential Mechanisms and Improvement Strategies. Food Front..

[223] Hsu C.C., Wang S.I., Yu S., Lin E.S., Wei J.C.C. (2025). Adherence to an Anti-Inflammatory Diet Is Associated with Lower Alzheimer’s Disease Mortality: A Modifiable Risk Factor in a National Cohort. J. Prev. Alzheimer’s Dis..

[224] Das D., Shruthi N.R., Banerjee A., Jothimani G., Duttaroy A.K., Pathak S. (2023). Endothelial Dysfunction, Platelet Hyperactivity, Hypertension, and the Metabolic Syndrome: Molecular Insights and Combating Strategies. Front. Nutr..

[225] Avilez-Avilez J.J., García-Aviles J.E., Ramírez-Carreto R.J., Salas-Venegas V., Guzmán-Ruiz M.A., Medina-Flores F., Königsberg M., Chavarría A., Gómez-González B. (2025). Progressive Blood–Brain Barrier Disruption in Sleep-Restricted Young Mice: Cellular Senescence and Neuroinflammation Crosstalk. Neurochem. Res..

[226] Cox B., Nicolaï J., Williamson B. (2023). The Role of the Efflux Transporter, P-Glycoprotein, at the Blood–Brain Barrier in Drug Discovery. Biopharm. Drug Dispos..

[227] Varghese S.M., Patel S., Nandan A., Jose A., Ghosh S., Sah R.K., Menon B., K V A., Chakravarty S. (2024). Unraveling the Role of the Blood-Brain Barrier in the Pathophysiology of Depression: Recent Advances and Future Perspectives. Mol. Neurobiol..

[228] Harris W.J., Asselin M.C., Hinz R., Parkes L.M., Allan S., Schiessl I., Boutin H., Dickie B.R. (2023). In Vivo Methods for Imaging Blood–Brain Barrier Function and Dysfunction. Eur. J. Nucl. Med. Mol. Imaging.

[229] Chen Y., He Y., Han J., Wei W., Chen F. (2023). Blood-Brain Barrier Dysfunction and Alzheimer’s Disease: Associations, Pathogenic Mechanisms, and Therapeutic Potential. Front. Aging Neurosci..

[230] Vetter J., Palagi I., Waisman A., Blaeser A. (2025). Recent Advances in Blood-Brain Barrier-on-a-Chip Models. Acta Biomater..

[231] Katt M.E., Shusta E.V. (2020). In Vitro Models of the Blood-Brain Barrier: Building in Physiological Complexity. Curr. Opin. Chem. Eng..

[232] Ingber D.E. (2022). Human Organs-on-Chips for Disease Modelling, Drug Development and Personalized Medicine. Nat. Rev. Genet..

[233] Zhou W., Xia S., Wang C., Yang Q., Verkhratsky A., Niu J. (2025). Critical Analysis of Translational Potential of Rodent Models of White Matter Pathology across a Wide Spectrum of Human Diseases. Cell Death Dis..

[234] Glassman P.M., Myerson J.W., Ferguson L.T., Kiseleva R.Y., Shuvaev V.V., Brenner J.S., Muzykantov V.R. (2020). Targeting Drug Delivery in the Vascular System: Focus on Endothelium. Adv. Drug Deliv. Rev..

[235] Zhong J., Gao R.R., Zhang X., Yang J.X., Liu Y., Ma J., Chen Q. (2025). Dissecting Endothelial Cell Heterogeneity with New Tools. Cell Regen..

[236] Sarvepalli S., Pasika S.R., Verma V., Thumma A., Bolla S., Nukala P.K., Butreddy A., Bolla P.K. (2025). A Review on the Stability Challenges of Advanced Biologic Therapeutics. Pharmaceutics.

[237] Lipska B.K., Weinberger D.R. (2000). To Model a Psychiatric Disorder in Animals: Schizophrenia as a Reality Test. Neuropsychopharmacology.

[238] Humer E., Probst T., Pieh C. (2020). Metabolomics in Psychiatric Disorders: What We Learn from Animal Models. Metabolites.

[239] Sarapultsev A., Komelkova M., Lookin O., Khatsko S., Gusev E., Trofimov A., Tokay T., Hu D. (2024). Rat Models in Post-Traumatic Stress Disorder Research: Strengths, Limitations, and Implications for Translational Studies. Pathophysiology.

[240] von Mücke-Heim I.A., Urbina-Treviño L., Bordes J., Ries C., Schmidt M.V., Deussing J.M. (2022). Introducing a Depression-like Syndrome for Translational Neuropsychiatry: A Plea for Taxonomical Validity and Improved Comparability between Humans and Mice. Mol. Psychiatry.

[241] Bao J., Chang C., Zhang Q., Saykin A.J., Shen L., Long Q. (2023). Integrative Analysis of Multi-Omics and Imaging Data with Incorporation of Biological Information via Structural Bayesian Factor Analysis. Brief. Bioinform..

[242] Moyaert P., Padrela B.E., Morgan C.A., Petr J., Versijpt J., Barkhof F., Jurkiewicz M.T., Shao X., Oyeniran O., Manson T. (2023). Imaging Blood-Brain Barrier Dysfunction: A State-of-the-Art Review from a Clinical Perspective. Front. Aging Neurosci..

[243] Andjelkovic A.V., Situ M., Citalan-Madrid A.F., Stamatovic S.M., Xiang J., Keep R.F. (2023). Blood-Brain Barrier Dysfunction in Normal Aging and Neurodegeneration: Mechanisms, Impact, and Treatments. Stroke.

[244] Yu M., Nie Y., Yang J., Yang S., Li R., Rao V., Hu X., Fang C., Li S., Song D. (2023). Integrative Multi-Omic Profiling of Adult Mouse Brain Endothelial Cells and Potential Implications in Alzheimer’s Disease. Cell Rep..

[245] Zhao Q., Zhang C., Zhang W., Zhang S., Liu Q., Guo Y. (2025). Applications and Challenges of Biomarker-Based Predictive Models in Proactive Health Management. Front. Public Health.

[246] Ren F., Wei J., Chen Q., Hu M., Yu L., Mi J., Zhou X., Qin D., Wu J., Wu A. (2025). Artificial Intelligence-Driven Multi-Omics Approaches in Alzheimer’s Disease: Progress, Challenges, and Future Directions. Acta Pharm. Sin. B.

[247] Herzog C.M.S., Goeminne L.J.E., Poganik J.R., Barzilai N., Belsky D.W., Betts-LaCroix J., Chen B.H., Chen M., Cohen A.A., Cummings S.R. (2024). Challenges and Recommendations for the Translation of Biomarkers of Aging. Nat. Aging.

[248] Kealy J., Greene C., Campbell M. (2020). Blood-Brain Barrier Regulation in Psychiatric Disorders. Neurosci. Lett..

[249] Sadat Razavi Z., Sina Alizadeh S., Sadat Razavi F., Souri M., Soltani M. (2025). Advancing Neurological Disorders Therapies: Organic Nanoparticles as a Key to Blood-Brain Barrier Penetration. Int. J. Pharm..

[250] Yazla E., Kayadibi H., Cetin I., Aydinoglu U., Karadere M.E. (2022). Evaluation of Changes in Peripheric Biomarkers Related to Blood Brain Barrier Damage in Patients with Schizophrenia and Their Correlation with Symptoms. Clin. Psychopharmacol. Neurosci..

[251] Hillmer L., Erhardt E.B., Caprihan A., Adair J.C., Knoefel J.E., Prestopnik J., Thompson J., Hobson S., Rosenberg G.A. (2022). Blood-Brain Barrier Disruption Measured by Albumin Index Correlates with Inflammatory Fluid Biomarkers. J. Cereb. Blood Flow Metab..

[252] Koh S.X.T., Lee J.K.W. (2014). S100B as a Marker for Brain Damage and Blood-Brain Barrier Disruption Following Exercise. Sports Med..

[253] Gayger-Dias V., Vizuete A.F.K., Rodrigues L., Wartchow K.M., Bobermin L., Leite M.C., Quincozes-Santos A., Kleindienst A., Gonçalves C.A. (2023). How S100B Crosses Brain Barriers and Why It Is Considered a Peripheral Marker of Brain Injury. Exp. Biol. Med..

[254] Janigro D., Mondello S., Posti J.P., Unden J. (2022). GFAP and S100B: What You Always Wanted to Know and Never Dared to Ask. Front. Neurol..

[255] Cash A., Theus M.H. (2020). Mechanisms of Blood–Brain Barrier Dysfunction in Traumatic Brain Injury. Int. J. Mol. Sci..

[256] Hosseinkhani B., Duran G., Hoeks C., Hermans D., Schepers M., Baeten P., Poelmans J., Coenen B., Bekar K., Pintelon I. (2023). Cerebral Microvascular Endothelial Cell-Derived Extracellular Vesicles Regulate Blood—Brain Barrier Function. Fluids Barriers CNS.

[257] Ramos-Zaldívar H.M., Polakovicova I., Salas-Huenuleo E., Corvalán A.H., Kogan M.J., Yefi C.P., Andia M.E. (2022). Extracellular Vesicles through the Blood–Brain Barrier: A Review. Fluids Barriers CNS.

[258] Wang Q., Huang X., Su Y., Yin G., Wang S., Yu B., Li H., Qi J., Chen H., Zeng W. (2022). Activation of Wnt/β-Catenin Pathway Mitigates Blood–Brain Barrier Dysfunction in Alzheimer’s Disease. Brain.

[259] Ko E., Kamm R.D. (2022). Neurovascular Models for Organ-on-a-Chips. Vitr. Models.

[260] Mulay A.R., Hwang J., Kim D.H. (2024). Microphysiological Blood-Brain Barrier Systems for Disease Modeling and Drug Development. Adv. Healthc. Mater..

[261] Reiner O., Sapir T., Parichha A. (2021). Using Multi-Organ Culture Systems to Study Parkinson’s Disease. Mol. Psychiatry.

